# Achieving high permeability and enhanced selectivity for Angstrom-scale separations using artificial water channel membranes

**DOI:** 10.1038/s41467-018-04604-y

**Published:** 2018-06-12

**Authors:** Yue-xiao Shen, Woochul Song, D. Ryan Barden, Tingwei Ren, Chao Lang, Hasin Feroz, Codey B. Henderson, Patrick O. Saboe, Daniel Tsai, Hengjing Yan, Peter J. Butler, Guillermo C. Bazan, William A. Phillip, Robert J. Hickey, Paul S. Cremer, Harish Vashisth, Manish Kumar

**Affiliations:** 1 Department of Chemical Engineering, The Pennsylvania State University, University Park, PA 16802 USA; 2 Department of Chemical Engineering, University of New Hampshire, Durham, NH 03824 USA; 3 Department of Chemistry, The Pennsylvania State University, University Park, PA 16802 USA; 4 Center for Polymers and Organic Solids, University of California at Santa Barbara, Santa Barbara, CA 93106 USA; 5 Department of Biomedical Engineering, The Pennsylvania State University, University Park, PA 16802 USA; 6 Department of Chemical and Biomolecular Engineering, University of Notre Dame, Notre Dame, IN 46556 USA; 7 Department of Material Science and Engineering, The Pennsylvania State University, University Park, PA 16802 USA; 8 Department of Civil and Environmental Engineering, The Pennsylvania State University, University Park, PA 16802 USA; 9 Present Address: Department of Chemistry, University of California, Berkeley, CA 94720 USA

## Abstract

Synthetic polymer membranes, critical to diverse energy-efficient separations, are subject to permeability-selectivity trade-offs that decrease their overall efficacy. These trade-offs are due to structural variations (e.g., broad pore size distributions) in both nonporous membranes used for Angstrom-scale separations and porous membranes used for nano to micron-scale separations. Biological membranes utilize well-defined Angstrom-scale pores to provide exceptional transport properties and can be used as inspiration to overcome this trade-off. Here, we present a comprehensive demonstration of such a bioinspired approach based on pillar[5]arene artificial water channels, resulting in artificial water channel-based block copolymer membranes. These membranes have a sharp selectivity profile with a molecular weight cutoff of ~ 500 Da, a size range challenging to achieve with current membranes, while achieving a large improvement in permeability (~65 L m^−2^ h^−1^ bar^−1^ compared with 4–7 L m^−2^ h^−1^ bar^−1^) over similarly rated commercial membranes.

## Abstract (web summary)

Synthetic polymeric membranes used for separations suffer from permeability-selectivity trade-offs. Here the authors demonstrate how a bioinspired pillar[5]arene artificial water channel embedded in a copolymer membrane can improve selectivity while still achieving high permeability.

## Introduction

Synthetic polymeric membranes, which are widely used in water purification, gas separations, chemical processing, and bioprocessing, suffer from a ubiquitous trade-off trend: high permeability leads to low selectivity and vice versa^1^. This trade-off manifests owing to the structural variations in state-of-the-art membranes, which typically use differences in molecular sizes to effect a separation. In the cases of angstrom-scale separations such as water desalination^2^ and gas separations^3^, where nonporous polymeric membranes operate via the solution-diffusion mechanism^4^, the variable size of the free volume elements through which diffusion occurs hampers membrane performance^1^. In porous membranes such as ultrafiltration and microfiltration, this trade-off is primarily due to the broad pore size distribution seen in commercial membranes^5^.

Biological membranes exhibit high permeability and high selectivity because they possess transmembrane proteins^6^ with well-defined channel pore sizes that synergistically combine size, charge, van der Waals, and specific binding interactions within the channels to enhance the transport of target species. For example, size exclusion is the major mechanism for water-over-solute selectivity of biological water channel proteins, aquaporins (AQPs)^7^. Water dipole reorientation through a series of specific hydrogen bonding steps is another selectivity mechanism that prevents protons from crossing the membrane^8^. AQPs have been incorporated into membrane matrices for applications in desalination and water purification^9^ because of their ability to maintain high permeability and selectivity. However, high costs, difficulties in fabrication, and low stability associated with membrane protein-based materials have hindered the large-scale applications of AQP-based membranes^9^. Concurrent with work on AQP-based membranes, an increase in the knowledge regarding the structure-function relationships of AQPs has inspired a series of artificial structures, including artificial water channels^10^ and carbon nanotube porins (CNTPs)^11,12^, which attempt to mimic the high water permeability and selectivity of AQPs. Artificial channels can be synthesized using simple chemistry, and are solvent compatible, thus allowing manufacturing techniques common in polymer processing to be applied^13^. More importantly, flexibility in design of the chemical structures of artificial channels further allows for specific functionalization to tailor their permeability^13,14^ and selectivity^15,16^. These precisely designed pore structures are ideal for membranes that can overcome the aforementioned permeability-selectivity trade-off of current commercial membranes.

**Fig. 1:**
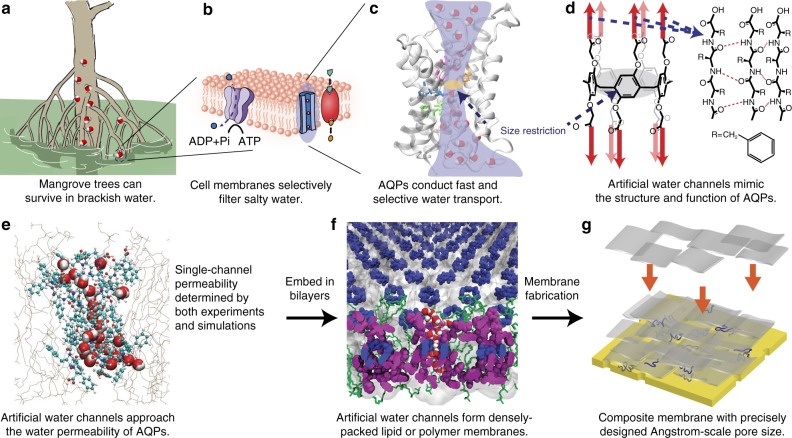
Biological inspiration and molecular design of highly selective and permeable water channels, their self-assembly, and translation to macroscale membranes. **a** Biological cell membranes (e.g., mangrove root cell membranes) are capable of efficiently and selectively transporting water. **b**, **c** This transport across membranes is mediated by transmembrane proteins including water channel proteins such as aquaporins (AQPs). **d**, **e** Artificial water channels, which have a similar hydrophobic outer surface and constrict to Angstrom-scale apertures at their narrowest points like AQPs, maintain high permeability and selectivity. **f**, **g** They also provide a route toward large-scale applications owing to their relatively simple synthesis and chemical and mechanical stability. With carefully designed self-assembly and membrane fabrication procedures, densely packed artificial water channel-based membranes provide molecular separation at the designed pore size while maintaining high permeability

In this paper, we present an inception-to-implementation description of highly permeable and selective polymer membranes consisting of well-defined, densely packed artificial water channels (Fig. 1). The design of the channel used in this work, peptide-appended pillar[5]arene (PAP) artificial water channel with a pore size of ~ 5 Å^13^, was inspired by AQPs found in cell membranes including in those of mangrove roots (Fig. 1a–d). To date, artificial water channels, including PAP channels^13^ and CNTPs^11,12^, have been primarily characterized in lipid bilayers^13–18^, which are not ideal for larger scale applications owing to their low chemical and mechanical stability^19^. As such, we first systematically investigated the molecular transport of water through PAP channels embedded in amphiphilic block copolymers (BCPs). BCPs form lipid-like bilayer structures^20^, and are more mechanically and chemically stable than lipids^20^, and offer customizable polymer types^21^, membrane thicknesses^20,21^ and terminal functional groups^22^. BCPs are being increasingly used for incorporating membrane proteins into bilayer or bilayer-like matrices for functional studies^23^, drug delivery^24^, and sensors^25^, and self-assembled ultrafiltration membranes^26^. We chose poly(butadiene)-*b*-poly(ethylene oxide) di-block copolymers (denoted as PB_n_-PEO_m_, see Supplementary Table 1, *n* and *m* indicate the number average degree of polymerization for each block) because they have been used to study membrane proteins and demonstrate unique self-assembly properties including the ability to assemble membrane proteins into two-dimensional crystals^27,28^. The water conductance of PAP channels in BCPs was determined to be in the range of AQPs’ (~10^8^–10^9^ water molecules s^−1^), confirmed by both experiments and simulations (Fig. 1e). We also studied the effect of physical hydrophobic mismatch, which is characterized by the difference between the bilayer thickness and channel length, and chemical hydrophobic mismatch, which is indicative of the differences in surface energy between the hydrophobic domains of the bilayers and the outer surface of the channels, on insertion of channels into lipid and polymer bilayers. PAP channels were then densely packed into two-dimensional sheets within PB-PEO polymers (Fig. 1f) through a controlled self-assembly process; and the composite membranes were synthesized by depositing these 2D sheets using a layer-by-layer technique on porous substrates. These membranes preserve the exceptional permeability and selectivity properties of PAP channels determined in molecular transport studies (Fig. 1g) with permeabilities and order of magnitude above corresponding commercial membranes while maintaining a close to ideal selectivity expected from a membrane with monodisperse 5 Å pores.

## Results

### Functional characterization of PAP channels in PB-PEO polymer membranes

**Fig. 2:**
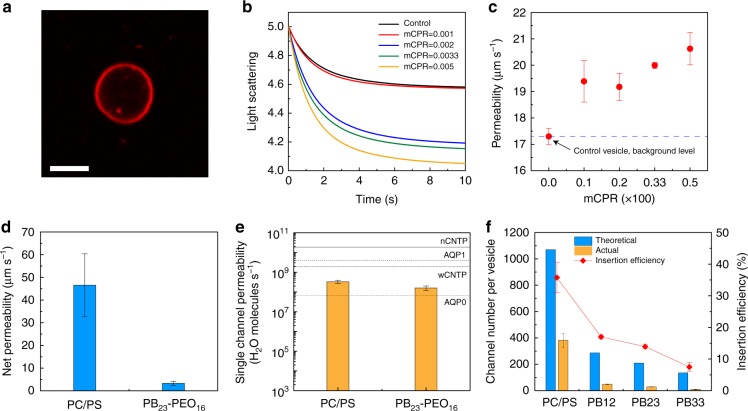
Peptide-appended pillar[5]arene (PAP) channels maintain an aquaporin-like single channel permeability in block copolymer bilayers. **a** Confocal fluorescence microscope image of a giant unilamellar vesicle showing the incorporation of fluorescently labeled PAP channels into PB23 polymersomes. Block copolymers were not labeled. Scale bar, 5 μm. **b** Representative light scattering traces of PB23 polymersomes with different molar channel-to-polymer ratios (mCPRs) after a rapid exposure to a hypotonic solution in a stopped-flow instrument. The hypotonic solution lacked the 100 mM PEG600 used to form the vesicles. **c** The water permeability of PAP channel-containing PB23 polymersomes formed with different mCPRs measured under hypotonic conditions. The dash line showed the background permeability level of a control PB23 vesicle. **d** Net water permeability of PAP channels in liposomes (4:1 (mol/mol) phosphatidylcholine/phosphatidylserine (PC/PS), molar channel-to-lipid ratio (mCLR) is 0.005), and in PB23 polymersomes (mCPR = 0.005) after subtraction of the background permeability of control vesicles shown in **c**. **e** Single channel water permeability of PAP channels in PC/PS liposomes and PB23 polymersomes. The permeabilities of AQP1, AQP0 and CNTP are from Zeidel et al.^30^, Saobe et al.^31^, and Tunuguntla et al.^12^, respectively. **f** The insertion efficiency of PAP channels in PC/PS liposomes and PB-PEO polymersomes was determined using fluorescence correlation spectroscopy (FCS) measurements. The calculation of insertion efficiency is described in detail in Supplementary Methods. Data shown are the average of triplicates with standard deviation

PAP channels could be incorporated into PB-PEO bilayers using the film rehydration method (Fig. 2a) previously used for incorporating artificial and biological water channels in lipid bilayers^13,27^. For PB_23_-PEO_16_ (PB23), vesicles with diameters from 200–300 nm were formed, as indicated by both transmission electron microscopy (TEM) and dynamic light scattering size measurements (Supplementary Fig. 1). After incorporating PAP channels into these unilamellar polymersomes at different molar channel-to-polymer ratios (mCPRs), the polymersomes were abruptly exposed to an inwardly directed osmotic gradient, the subsequent swelling of the polymersomes led to greater destructive interference and a concomitant decrease in the scattering intensity especially at larger angles. This is because vesicles with a size comparable to the wavelength of light, i.e., sizes > 1/10 the wavelength of light, stop acting like point particles and show a decreasing trend in scattering intensity with increasing volume^29^ at the scattering angle used for measurements (90**°**). The light scattering signals decreased faster at higher mCPRs (Fig. 2b), indicative of faster volumetric expansion and thus higher water permeance in the presence of channels (Fig. 2c). The PB23 vesicles, at an mCPR of 0.005, had a net permeability of 3.3 ± 0.9 μm s^−1^ (mean ± s.d., *n* = 3, Fig. 2d) after subtracting the background shown in Fig. 2c.

The number of PAP channels per polymersome was counted using a fluorescence correlation spectroscopy (FCS)-based technique reported previously^13^ and illustrated in Supplementary Fig. 2 and Supplementary Methods. PAP channels were first tagged with a fluorophore, tetramethylrhodamine cadaverine^13^. Polymersomes with labeled channels were subjected to FCS analysis before and after detergent solubilization, creating an autocorrelation function for the vesicles prior to solubilization and another for the solubilized micelles containing PAP channels (Supplementary Fig. 2). A double-species model (used to differentiate labeled PAP channels from residual free fluorophores) was employed to fit each of the autocorrelation curves and to obtain the number of fluorescent vesicles (*N*_Vesicle_) and solubilized PAP micelles (*N*_Micelles_). The number of the inserted channels per vesicle (*N*_Channels_) was calculated as the ratio *N*_Micelles_/*N*_Vesicle_. The number of inserted channels per vesicle at an mCPR of 0.005 was ~ 30 (Supplementary Fig. 3).

We compared the water conductance of PAP channels in lipid and PB23 membranes (Fig. 2d). At a molar channel-to-lipid ratio of 0.005 (mCLR = 0.005), the net permeability of PAP channels in lipid vesicles was 46.6 ± 13.8 μm s^−1^, approximately one order of magnitude higher compared with that in PB23 polymersomes (3.3 ± 0.9 μm s^−1^) at an mCPR of 0.005. However, the number of inserted PAP channels in the lipid system were also found to be ~10 times higher than that in the PB23 vesicles (Supplementary Fig. 3). Combined, the FCS and the stopped-flow permeability data showed that the single channel water permeability of PAP channels in PB23 vesicles was 1.6 ± 0.4 × 10^8^ H_2_O molecules s^−1^ (Fig. 2e), which was lower by a factor of ~ 2 compared with that in liposomes (3.3 ± 0.6 × 10^8^ H_2_O molecules s^−1^)^13^ but within the range of AQPs and CNTPs (~10^8^−10^10^ water molecules s^−1^)^12,30,31^. Note that in this study membrane permeability is presented in the units of flux at applied osmotic gradient (μm s^−1^) for bilayer membranes or the industry standard (L m^−2  ^h^−1^ bar^−1^, denoted as LMH bar^−1^) for synthetic membranes (1 μm s^−1^ is equivalent to 2.75 × 10^−3^ LMH bar^−1^, see Supplementary Methods)^32^. Single channel permeability is reported as volumetric flow rate (cm^3^ s^−1^) or number of water molecules per s (3 × 10^−14^ cm^3^ s^−1^ is equivalent to 10^9^ water molecules s^−1^). These units were chosen for easy comparison with literature values in the two distinct fields of membrane biophysics and synthetic membrane science.

In addition to PB23, we also incorporated channels in PB_12_-PEO_9_ (PB12) and PB_33_-PEO_24_ (PB33) BCPs with different polymer lengths (Supplementary Table 1). Although the selected BCPs had a hydrophilic volume fraction “*f*_hydrophilic_” within the threshold expected to form vesicles (0.35 ± 0.10, Supplementary Table 1)^33,34^, the rehydration of OH-terminated PB12 resulted only in micelles (Supplementary Fig. 4); COOH functionalized PB12 produced a vesicle and micelle mixture (Supplementary Fig. 5)^35^; and PB33 polymers formed worm-like structures^34^ with < 10% proportion of vesicles (Supplementary Fig. 6). After size exclusion chromatography to remove the non-vesicular fractions, COOH functionalized PB12, OH-terminated PB23 and OH-terminated PB33 vesicles were used to study channel insertion efficiency (Supplementary Fig. 3 and 7). Insertion efficiency is defined as the actual channel number per vesicle determined by FCS divided by the theoretical insertion number, for which we assumed all PAP channels added to the reconstitution mixture were embedded into the vesicle bilayers (Fig. 2f, see Supplementary Methods). In lipid vesicles at an mCLR of 0.005, the number of PAP channels per liposome (383 ± 52 channels in a vesicle of ~ 160 nm in diameter) corresponded to an insertion efficiency of 35.7 ± 4.9%. The number decreased to 17.0 ± 0.6%, 13.9 ± 0.6% and 7.5 ± 1.5% for PB12, PB23, and PB33 vesicles, respectively.

The insertion of PAP channels was less favorable in PB-PEO polymers than lipids, and was dependent on hydrophobic block length and chemical hydrophobic mismatch with the polymer. From the perspective of physical compatibility, the insertion ability in PB polymers decreased with increasing polymer hydrophobic block lengths. PB12, PB23, and PB33 polymers have a hydrophobic bilayer thickness of 5.1 ± 0.6 nm, 6.0 ± 0.5 nm, and 7.4 ± 0.5 nm, respectively, as estimated from Cryo-TEM (Supplementary Fig. 8), which were close to the thicknesses estimated from literature (Supplementary Table 1 and Supplementary Fig. 9, see Supplementary Methods). The physical mismatch with the height of PAP channels (~4 nm) further supported the observed trend for PAP channels in PB-PEO BCPs. The chemical compatibility between the channels and the bilayer hydrophobic block was quantified by a water soluble conjugated oligoelectrolyte, 4,4’-bis(4’-(*N*,*N*-bis(6”-(*N*,*N*,*N*-trimethylammonium)hexyl)amino)-styryl)stilbene tetraiodide (DSSN+). This molecule was used to probe relative hydrophobicity of bilayer hydrophobic blocks. DSSN+ has a D-π-D structure and undergoes intramolecular charge transfer that is influenced by the solvent environment^36^, and can be used to probe the hydrophobicity of self-assembled membranes as shown in a recent study^37^. The insertion of DSSN+ into lipids leads to a blue shift of the emission due to the lower-polarity inner core of a lipid bilayer compared with aqueous environment (Supplementary Fig. 10)^36^. The lipid induced a larger blue shift (57.3 ± 0.9 nm) than PB23 BCPs (8.0 ± 1.3 nm) in DSSN+ emission (Supplementary Fig. 11), indicative of a more hydrophobic environment in lipid than in PB polymers. When PAP channels were incorporated into phosphatidylcholine/phosphatidylserine liposomes, the blue shift increased from 57.3 ± 0.9 nm to 64.0 ± 0.0 nm, whereas this shift was from 8.0 ± 1.3 nm to 18.0 ± 0.0 nm for PB23 polymersomes. The relative shift was 28.1 and 89.5% for pure liposomes and PB23 polymersomes, respectively, implying the phenylalanine arms of PAP channels were closer in hydrophobicity to the lipid membrane environment rather than the PB membrane environment, resulting in a higher driving force for spontaneous insertion^38–40^ in the hydrophobic phospholipid core. We concluded that the insertion of PAP channels was more favorable in lipid than in PB-PEO membranes from the perspective of chemical compatibility. Thus, opportunities exist to develop other polymer chemistries that may be more compatible with PAP channels.

### Molecular dynamics simulations of PAP channels in BCP membranes

We performed several independent long timescale molecular dynamics (MD) simulations of a single PAP channel in two BCP membranes (PB12 and PB23) and one lipid membrane (1-palmitoyl-2-oleoyl-*sn*-glycero-3-phosphocholine, POPC) (Supplementary Table 2). For BCP simulations, the polymer chains were first placed in a di-block arrangement and simulations were conducted until the pristine membranes reached equilibration (Supplementary Fig. 12). The hydrophobic layer thickness of PB12 and PB23 membranes upon equilibration were 3.7 ± 0.1 nm and 5.4 ± 0.1 nm, respectively. These values are consistent with those measured in experiments (Supplementary Fig. 8). We used these equilibrated BCP membranes for PAP-embedded simulations.

**Fig. 3:**
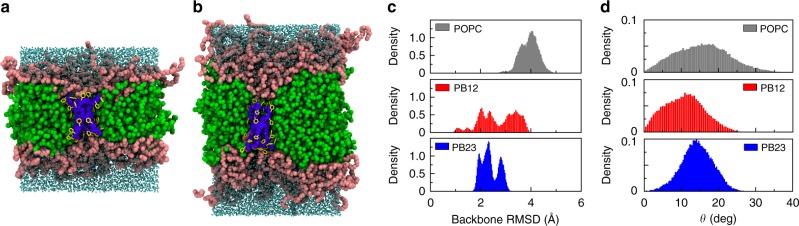
MD simulations of PAP channels in bilayers demonstrate adaptation to PAP length, and unique restraining effects of polymers. Snapshots of single PAP channel conformation in PB_12_-PEO_9_ (**a**) and PB_23_-PEO_16_ (**b**) bilayer membranes at *t* = 200 ns in each MD simulation. The hydrophobic PB chains are shown in green and the hydrophilic PEO chains are shown in pink spheres. The water molecules are cyan. The PAP channel backbone is blue with yellow aromatic rings on the side chains. **c** The root-mean-squared-deviation (RMSD, with respect to its initial conformation) of the PAP channel backbone in POPC membranes was larger than that in either PB12 or PB23 membranes. The smaller RMSD value for PB polymers suggested that the channel’s ‘peptide-arm’ movements were more constrained in block copolymer membranes. **d** The averaged angle orientation during the simulation showed that the PAP channel (an angle of 0° indicates a conformation perpendicular to the membrane surface and an angle of 90° indicates a conformation parallel to the membrane surface) preferred to keep an averaged angle of ~ 15° in three types of the membranes. The broader angle distribution in POPC membranes indicated that PAP channels were more flexible with more peptide-arm movement flexibility in lipid membranes than in PB-PEO membranes. Data shown are averages over triplicate simulations

Simulations demonstrated that the dynamics of a single PAP channel are different in the three types of membranes (Supplementary Figs. 13–15). Given that the hydrophobic thickness (~3.7 nm) of the PB12 membrane matches the height of the PAP channel (~4 nm), a single PAP channel spanned the PB12 membrane (Fig. 3a) without significant membrane deformation. However, the PB23 membrane near the channel was observed to significantly change its thickness in order to accommodate the PAP channel (Fig. 3b). This phenomenon has been observed in the case of membrane proteins and predicted to be important for the stabilization of membrane proteins in BCP membranes^23^. Channel dynamics were quantified by mean-squared-displacement (MSD) (Supplementary Fig. 16) and root-mean-squared-deviation (RMSD) (Supplementary Fig. 17) calculations. MSD represents the diffusion of a single channel within the hydrophobic core of membranes and we found that the lateral movement in the plane of each membrane dominated the diffusion (Supplementary Fig. 16). Both lateral and vertical diffusivities of a single PAP channel in BCP membranes were lower than in lipid membranes (Supplementary Fig. 16 and Supplementary Table 3). RMSD is a measure of the configuration change of the channel itself within membranes (Fig. 3c and Supplementary Fig. 17). This self-mobility showed a decrease from POPC to PB12 and then to PB23. The outcome of this mobility and the interaction with membranes was the tilting of PAP channels at an angle of ~15–20° (Fig. 3d, Supplementary Fig. 18 and Supplementary Table 3)^11,13^. Similar to MSD and RMSD, the distribution of angles became narrower from POPC, to PB12 and to even thicker PB23 membranes, indicating that PAP channels were more constrained and stable, as in the phenylalanine peptide “arms” were less mobile in polymeric membranes.

The permeability of a single PAP channel was measured in PB-PEO membranes and compared with the lipid reference using a widely used collective diffusion model (Supplementary Fig. 19)^41^. The average permeability of a PAP channel in POPC, PB12 and PB23 was 1.8 ± 0.1 × 10^9^ water molecules s^−1^, 6.0 ± 0.1 × 10^8^ water molecules s^−1^ and 8.3 ± 0.1 × 10^8^ water molecules s^−1^, respectively. These values are of the same order of magnitude and reflect a similar difference between lipid and polymer systems for channels as determined in the experimental data at 3.3 ± 0.6 × 10^8^ water molecules s^−1^ for lipids and 1.6 ± 0.4 × 10^8^ water molecules s^−1^ for PB23, respectively.

### Self-assembly of PAP channels in PB-PEO membranes

**Fig. 4:**
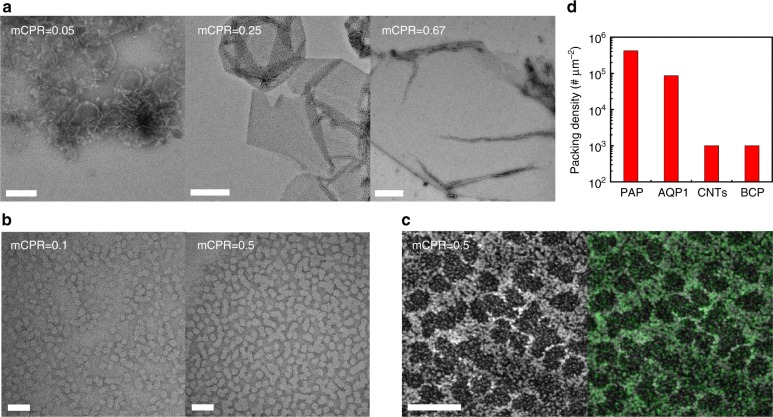
PAP concentration influences the morphology of self-assembled PAP-BCP aggregates and PAP channels segregate into rafts. **a** Negative-stain TEM images show the representative morphologies of aggregates formed at different molar channel-to-polymer ratios (mCPRs). Increasing mCPRs from 0.05, 0.25 to 0.67 resulted in morphology transitions from small vesicles to large vesicles, and finally to flat membranes. Scale bars, 0.1 μm, 0.5 μm, and 1 μm, from left to right, respectively. **b** TEM images at high magnification show the microphase separation in PB12 membranes. Uranyl formate selectively stained PB-PEO. The brighter unstained microdomains of 20–30 nm are PAP channels and the density of these domains increased from low (left, mCPR = 0.1) to high (right, mCPR = 0.5) channel concentrations. **c** High-resolution scanning TEM (STEM) and energy dispersive spectroscopy (EDS) map supported the microphase separation hypothesis. The left is the high-angle annular dark-field (HAADF) image. The right is the HAADF overlaid with uranyl map (green). The uranyl map aligned well with the PB polymer enriched region. Scale bar, 50 nm. **d** The packing density of PAP channels in PB12 membranes was ~4.2 × 10^5^ μm^−2^, more than two orders of magnitude higher that packing density of CNTs (0.1~2.5 × 10^3^ μm^−2^)^44–46^ and block copolymer templated nanopore membranes (~10^3^ μm^−2^)^47^. Scale bar, 40 nm

Though PB12 BCPs form micellar structures in aqueous solutions^27^, they can produce highly ordered two-dimensional crystals with membrane proteins such as aquaporin-0 (AQP0)^27^ and outer membrane protein F^28^ after a controlled self-assembly process. Because of the similarity of the bilayer thickness to the length of the PAP channels, we hypothesized that they could be packed with high density in PB12 membranes. We performed dialysis of ternary PB12 BCP/PAP channels/detergent mixtures and allowed the detergent to be removed slowly through its critical micelle concentration, which has been shown to be critical for efficient membrane protein insertion^27,42^. PAP concentration had a significant impact on the morphology of PAP channel/PB12 aggregates after detergent removal. As observed by negative-stain TEM (Fig. 4a), PB12 formed small vesicles at an mCPR of 0.05, large vesicles at an mCPR of 0.1 and large flat sheets at higher mCPR ratios. These transitions in morphology demonstrate that increase in PAP channel density in the channel-BCP binary system changed bilayer curvature and resulted in the formation of flat membranes. In contrast to the uniform membrane with packed arrays formed by PAP channels in lipids^13^, PAP channels packed into PB12 membranes exhibited microphase separation as visualized by electron microscopy after negative staining by uranyl formate (Fig. 4b). The uranyl-stained PB-PEO BCPs implied the brighter domains of 20–30 nm in diameter were PAP aggregates and the number of the unstained domains per unit area also increased at higher mCPR ratios (Fig. 4b). Energy filtered TEM (Supplementary Fig. 20) and energy dispersive spectroscopy (EDS) (Fig. 4c) mapping confirmed that the microphase separated domains had enriched nitrogen species that are only present in PAP channels and not in PB-PEO BCPs.

Considering that the entire cross-sectional area of a PAP channel is ∼1.5 nm^2^,^43^ the packing density of PAP channels in PB12 membranes at an mCPR of 0.5 (Fig. 4c, right panel) was estimated to be ~4.2 × 10^5^ μm^−2^, more than two orders of magnitude higher that packing density of carbon nanotubes (CNTs) (0.1~2.5 × 10^3^ μm^−2^)^44–46^ and block copolymer templated nanopore membranes (~10^3^ μm^−2^)^47^ (Fig. 4d). The microphase separation could be attributed to the less favorable interaction between PAP channels and PB12 BCPs compared with those seen between PAP channels and lipids but does not seem to affect the overall packing density of channels, which is of similar magnitude to that measured in lipids. We further investigated the lateral diffusion of PAP channels at high concentration in BCPs using fluorescence recovery after photobleaching (FRAP). The FRAP recovery curve of the labeled PAP channels showed two different kinetic regimes: a fast recovery (Supplementary Fig. 21, inset, ~ 40% mobile fraction) with a larger diffusion coefficient of 0.086 μm^2^ s^−1^, and a long recovery (Supplementary Fig. 21, ~34% mobile fraction) with a smaller diffusion coefficient of 0.0069 μm^2^ s^−1^. The remaining 26% was the immobile fraction probably owing to the interaction between the labeled channels and the support. Diffusion in both the fast and slow regimes was found to be slower than the rate of single channel diffusion determined by MD simulations (Supplementary Table 3), which is likely a result the raft formation suggested by self-assembly experiments. These experimental results combined with simulations could provide insight into the dynamic behaviors of PAP channels and how artificial transmembrane channels stabilize themselves in synthetic polymer membranes.

### PAP[5] channel 2D sheet composite membranes

**Fig. 5:**
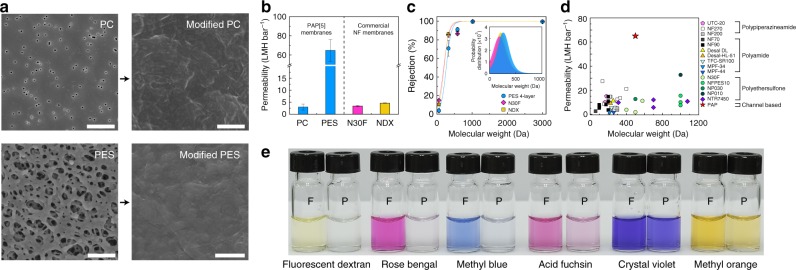
2D sheets of PAP[5] channels could be assembled into composite membranes and demonstrated enhanced performance compared with current commercial membranes. **a** SEM images showed ~ 100% coverage of PAP[5] 2D sheets on PC and PES membranes after three and four cycles of layer-by-layer deposition, respectively. Scale bar, 1 μm. **b** After three and four cycles of deposition on PC and PES membranes, the permeabilities were 3.0 ± 1.2 LMH bar^−1^ and 64.8 ± 11.3 LMH bar^−1^, respectively, (mean ± s.d., *n* = 3). The permeability of the modified PES membranes is approximately one order of magnitude higher that of commercial nanofiltration membrane N30F (3.4 ± 0.3 LMH bar^−1^) and NDX (4.6 ± 0.1 LMH bar^−1^) with a similar molecular weight cutoff (MWCO). **c** The molecular weight cutoff (MWCO) was ~ 450 Da, 370 Da, and 360 Da for the modified PES membrane, commercial N30F and NDX membranes, respectively, as determined from filtration of dyes of various molecular weights. The molecular weight cutoff probability distributions derived from fitting to a sigmoidal model showed the standard deviation (*σ*) of modified PES (132 Da) membranes was similar to that of N30F (146 Da) and NDX (125 Da). The pore size distribution is tighter for the PES membrane with a *σ*/MWCO ratio of 0.29 compared with 0.35–0.39 for the commercial membranes. The measured rejection values were corrected using a concentration polarization model and all the cutoff data were fit to a sigmoidal function, as described in Supplementary Methods. **d** A comparison of PAP[5] channel-based membrane to other commercial NF membranes shows that within the cutoff range (400~500 Da), the channel-based membrane has one order of magnitude higher permeability and in general is several times higher that of commercial nanofiltration membranes across the complete range of available data on such membranes from literature^50–64^. **e** Photographs of feed (F) and permeate (P) containing different dye molecules (decreasing MW from left to right in image) for modified PC membranes. Both positively and negatively charged dyes had similar rejections as shown in Supplementary Fig. 27. Data shown are the average of triplicate measurements with standard deviation

PAP channels formed micron sized 2D sheets or giant collapsed vesicles (Fig. 4), the large aspect ratio of which made them ideal for membrane fabrication. Each PAP channel has five carboxylic acid groups at each end of the channel. We functionalized PB12 BCPs with carboxylic groups so that the final 2D sheets were fully carboxylate terminated. A modified layer-by-layer method was then used to immobilize these 2D sheets onto polycarbonate track-etched membranes (PC) and polyethersulfone microfiltration (PES) support membranes with the aid of polyethyleneimine (PEI) (Fig. 1g)^48^. After three and four cycles of modification, 2D sheets achieved ~ 100% coverage on PC and PES membranes, respectively as observed by scanning electron microscopy (SEM, Supplementary Figs. 22, 23). The sharp edges of these sheets can be observed in the SEM images at a higher magnifications (Fig. 5a and Supplementary Fig. 24). The permeability gradually decreased with the increasing cycles of modification (Supplementary Fig. 25), and reached 3.0 ± 1.2 LMH bar^−1^ and 64.8 ± 11.3 LMH bar^−1^ for completely covered PC and PES membranes, respectively (Fig. 5b). A series of charged low molecular weight dyes were selected for conducting solute rejection tests. We first performed a filtration test on PEI-modified control membranes over a period of 60 min to exclude the adsorption effect contributed by polyelectrolytes (Supplementary Fig. 26)^49^ and a similar protocol was then followed with each of the selected dyes. The dye rejection properties improved as the number of depositions increased (Supplementary Fig. 26). The molecular exclusion limit of PC and PES membranes were ~560 Da and ~450 Da when completely covered with PAP 2D sheets, respectively (Fig. 5c), which is very close to the designed PAP pore, which has an exclusion limit of ~500 Da^13^. This size range is significant as only two commercial membranes exist with a MWCO ~ 500 Da (Fig. 5d) with the entire range of NF membranes reported in literature shown in Fig. 5d spanning 200–1000 Da and exhibiting an average permeability of ~10.0 ( ± 6.6) LMH bar^−1^^50–64^. The molecular weight cutoff probability distributions after fitting the solute exclusion data to a sigmoidal curve model showed that the composite PES membrane presented a sharper pore size distribution (based on the ratio of standard deviation to average MWCO) compared with two commercial membranes of MWCO ~ 400–500 Da: N30F^53^ and NDX (Fig. 5c), which were also tested using the same dyes. The composite membranes also showed a consistent molecular weight cutoff trend with both positively and negatively charged dyes (Fig. 5e and Supplementary Fig. 27). A series of steered MD simulations were conducted on the selected dye molecules, Methyl Orange (MO, 328 Da) and Rose Bengal (RB, 1018 Da), to validate the experimentally observed selectivity of the channel (Supplementary Fig. 28). We tested the conjecture by “pulling” each molecule through the central ring of PAP for 10 times and calculating the averaged potential of mean force (PMF) as a parameter to evaluate the ability of each molecule to be transport through the channel. We observed that MO could be pulled through the channel with relatively small values of PMF ( < 100 kcal mol^−1^), whereas RB required significantly greater PMF values ( > 1500 kcal mol^−1^). During steered MD simulations, the RB molecule remained stuck in the central ring until the pulling force was sufficient to deform both the molecule and PAP channel central ring enough to pull the molecule through, as shown in Supplementary Movies 1 and 2. The existence of significantly high free-energy barriers indicate that the transport of RB would not occur naturally. This series of simulations agree well with the diameter of PAP channels, earlier molecular transport studies^13^, and the molecular weight cutoff experiment of channel-based membranes in the filtration experiments. In addition, the channel-based membranes were also able effectively separate a mixture of methyl blue (800 Da) and MO (328 Da) as shown in Supplementary Fig. 29, while the commercial NF membranes N30F and NDX with a MWCO of 400–500 Da failed to achieve a clear molecular separation.

## Discussion

The composite membranes (especially modified PES membranes with porosity one order of magnitude higher than that of PC membranes) had a permeability of ~60 LMH bar^−1^ and a cutoff of 450 Da. In order to confirm the contribution of channels to molecular exclusion, we first performed a block copolymer control membrane test without PAP channels. Because the PB_12_-PEO_8_ only forms micelles or small vesicles during aqueous based self-assembly (Supplementary Fig. 5)^27^, synthesizing a control membrane using aqueous self-assembly alone^9^ was found to be excessively difficult. As an alternative, we adopted a solvent casting based method (Supplementary Fig. 30 A, see Supplementary Methods)^10,65^ and fabricated a fully covered block copolymer control membrane (Supplementary Fig. 30C). The control membrane displayed a low permeability (~0.1 LMH bar^−1^), and almost complete rejection (~96%) of the smallest dye used in this work (MO, 328 Da). This indicates that the high water transport permeability and the MWCO of ~500 Da are attributed primarily to the transport characteristics of the PAP channels, and not from the BCPs themselves. Second, the *d*-spacing between laminar 2D sheets was determined by X-ray diffraction as conducted in similar studies for laminar graphene oxide membranes^66^. This *d*-spacing was found to be 1.5 nm (Supplementary Fig. 31) for wet membranes, a spacing that is expected to allow a globular molecule of ~1 kDa to be accommodated^67^. This result indicates that the rejection from our membranes was mainly contributed by normal flow through PAP channels within these 2D sheets rather than the lateral flow observed between two sheets and then transverse flow between the sheets as in laminar graphene oxide and graphene membranes^68^. In addition, we evaluated the effects of a background electrolyte on the rejection of dyes. This was done to assess the potential contributions of electrostatic interactions to the solute rejection mechanism. The filtration experiments were performed at various ionic strengths. At the highest values of ionic strength the Debye length (*λ*_D_) of the solutions became comparable or even smaller than pore diameter of the PAP channel (Supplementary Fig. 32). At this limit, the electrostatic interactions between the membrane and solutes are effectively screened and electrostatic repulsion does not contribute significantly to the solute rejection mechanism^69^. As shown in Supplementary Fig. 32, both RB and acid fuchsin demonstrated minimal changes in rejection over this wide range of ionic strengths. This observation strongly indicates that the electrostatic interactions between solute and membrane do not contribute significantly to the solute rejection mechanisms of these channel-based membranes. The aforementioned experiments demonstrated that the channel-based size exclusion is the major transport mechanism for our composite membranes.

The permeability values measured for composite membranes were of the same order of magnitude as the values calculated from osmotic permeability measurements from stopped-flow experiments (see Supplementary Methods), with the measured permeability of composite membranes ~2–3 times larger. Given the uncertainties and differences in testing at these two very different scales (nm^2^ vs cm^2^ scale) and techniques (osmotic shock vs. pressure driven flow), we consider this agreement excellent. In terms of the rejection profile, the MWCO curve was sharper than those of commercial membranes (Fig. 5c). The obtained sigmoidal model does not completely rule out that some transport could occur through channels or defects of sizes different than the 5 Å pore size of the channels across these composite membranes but nevertheless indicates excellent performance of these first generation membranes. Possible defect mechanisms may include transport through the interlayer of the layer-by-layer structure, leakage through gaps between the block copolymer and the channels, diffusion through the block copolymer matrix itself, and leakage through uncovered substrate pores. During our fabrication, we tried to minimize leakage to the best of our ability. The *d*-spacing analysis demonstrated the rejection was mainly from the normal flow through PAP pores, but the lateral flow between layers could still have contributed to the overall flow. We also performed multiple layer-by-layer depositions and post chemical crosslinking to enhance the membrane integrity. In the long term, a fully cross-linked polymer matrix with better physical and chemical compatibility with channels is desired, and the fabrication process could be further optimized. However, owing to our ability to generate close to defect-free composite membranes, the permeability of the current PAP channel-based membrane is several times higher than current commercial nanofiltration and ultrafiltration membranes with MWCOs from 200 Da to 1 kDa (Fig. 5c, d)^53,70^, and its angstrom-scale separation was also demonstrated to be significantly enhanced over that of those commercial NF membranes as seen from example dye mixture separation experiments (Supplementary Fig. 29).

In terms of salt rejection, although PAP channels showed a similar order of magnitude ion selectivity (K^+^/Cl^−^ ratio of 10, corresponding to a permselectivity of 0.83)^18^ from patch clamp experiments based on anion exclusion as reported in a recently published CNTP paper^12^ (K^+^/Cl^−^ ratio of 80, corresponding to a permselectivity of 0.98), we did not observe high NaCl rejection during hydraulic pressure assisted filtration tests predicted by this recent study despite having a similar functionalization degree at the pore entrance. This difference is indicative of challenges in translating molecular transport properties, especially those from patch clamp experiments for solute transport on channels, to filtration performance. We hypothesize that artificial channels with smaller pore sizes than 5 Å may be needed for complete rejection of salts in a membrane context. We also recognize the possibility of imperfections in the polybutadiene domain of the polymer, particularly where they surround the channel leading to leakage of small solutes such as salts. A better understanding of polymer channel physical and chemical mismatch may allow selection and use of polymers that may be less susceptible to these possible defects.

In summary, we have fabricated a completely synthetic biomimetic membrane that consists of densely packed PAP artificial water channels. The resulting membrane maintained high permeability and, more importantly, maintained selectivity expected from the molecular design of these PAP channels. This approach of combining versatile supramolecular chemistry with careful self-assembly and membrane fabrication, could allow membranes with precisely designed angstrom-scale pore size to be constructed for next-generation liquid and gas separations.

## Methods

### Additional details provided in Supplementary Information

Supplementary Methods provide detailed information about materials and methods utilized in the current paper, including synthesis of PAP channels and BCPs, preparation of lipid and polymer vesicles, light-scatting based water transport, giant unilamellar vesicles (GUVs), labeling procedure, FCS, DSSN + experiments, self-assembly of PAP channel/polymer aggregates and TEM imaging, FRAP, unit conversion between osmotic permeability and traditional membrane permeability in filtration, simulation, and fabrication and characterization of PAP 2D sheets based membranes.

### Preparation of vesicles and permeability measurement

Polymersomes and liposomes were prepared by using the film rehydration method. PAP channels were added to BCPs or 4:1 (mol/mol) phosphatidylcholine/phosphatidylserine mixture in CHCl_3_. The mixture was gently dried in a rotary evaporator followed by high vacuum treatment to remove all solvent. The formed film was rehydrated with 1 ml buffer containing 10 mM Hepes, 100 mM PEG600, and 0.01% (w/v) NaN_3_ at pH of 7. The suspension was extruded through 0.2 μm track-etched membranes at least 10 times to obtain monodisperse and unilamellar vesicles, after incubation on a stir plate at 4 °C overnight. The size of the vesicles was measured using dynamic light scattering. The water permeability tests of polymer and lipid vesicles were conducted on an SF-300X stopped-flow instrument (KinTek Corp., PA) at 10 °C, where vesicles were rapidly exposed to a hypotonic osmolyte (10 mM Hepes and 0.01% (w/v) NaN_3_ at pH of 7). For DSSN + experiments, 5% (mol/mol) DSSN + was added into lipid and polymer vesicles during rehydration. After the final vesicle concentrations were diluted to 3 mg ml^−1^, these vesicles were incubated at 4 °C overnight. The UV-vis absorbance and emission spectra were scanned on a micro-plate reader.

### Channel labeling and related analysis

PAP channels were labeled with a rhodamine based fluorophore using a cross-linker dicyclohexylcarbodiimide^13^. The labeled channels were used to count the channel number per vesicle in FCS experiments, visually confirm the channel insertion into polymersomes using GUVs technique and study the diffusion properties of PAP channels in lipid or polymer bilayers in FRAP experiments.

### Self-assembly of channel/polymer aggregates

PAP channel/lipid aggregates were produced by a slow dialysis procedure^27^. First, PB12 or carboxylic PB12 copolymers and PAP channels were dissolved in CHCl_3_ and mixed at different mCPRs. After evaporating CHCl_3_, the film was dissolved in 60 μl rehydration buffer initially containing 4% (w/v) *n*-octyl-β-D-glucoside (OG). The PAP channel/polymer mixture was transferred into dialysis buttons with a 12-kDa cutoff membrane, where the final polymer concentration was 1 mg ml^−1^. The detergent concentration (initially 4%, w/v) was gradually lowered by doubling the dialysis buffer volume with detergent-free buffer every 8 h until the OG concentration in the dialysis buffer reached 0.25% (w/v). The dialysis buffer was then replaced with detergent-free buffer three times every 8 h. The polymersomes and PAP channel/BCPs aggregates were imaged by conventional TEM, Cryo-TEM, energy filtered TEM and scanning TEM with EDS map.

### Simulation methods

We conducted all MD simulations using NAMD^71^ and used VMD^72^ for visualization and analyses. The temperature and pressure in simulations were controlled at 303 K and 1 atm, using a Langevin thermostat and a Nose-Hoover barostat, respectively. We used the latest version of CHARMM force-field to simulate water, lipids, and polyethylene oxide^73^. The parameters used to simulate the PAP channel were identical to a previous study^13^. The necessary polymer chains for BCP membranes were created by first generating monomers using the molefacture plugin in VMD. Molefacture generates a pdb/psf pair and a topology file for each monomer. We then patched monomers together using VMD’s psfgen tool to form BCP chains of desired lengths. An existing patch was used to generate PEO chains, but new patches were built to join PB polymer blocks as well as to combine PB blocks with PEO blocks. All missing parameters for PB blocks and PB-PEO linkage were generated iteratively using the Force Field Toolkit (FFTk) plugin in VMD in combination with Gaussian quantum chemistry software. Specifically, to create PB blocks of any length, we parameterized a PB chain containing two monomers for which Lennard-Jones non-bonded parameters were assigned from existing parameter files in the CHARMM force-field. Gaussian was then used to optimize the geometry and to assign charges on all atoms. The missing parameters were generated and optimized using FFTk plugin in VMD and Gaussian software. All optimized CHARMM-compatible parameters for block copolymers are reported elsewhere^74^.

We used the membrane builder plugin in VMD to generate the POPC membrane. The POPC membrane used in our simulations was 70 Å × 70 Å in size. However, BCP membranes were generated by first constructing a single polymer chain of desired length for both PB and PEO blocks. The two independent chains were then connected using a patch, the parameters of which were optimized as described above. Each connected PB-PEO chain was then replicated and placed in a regular 10 × 10 grid pattern over an area of 80 Å × 80 Å (PB12) and 9 × 9 grid pattern over an area of 80 Å × 80 Å (PB23). All BCP membranes were arranged in a di-block configuration (Supplementary Fig. 12) prior to equilibration. Pristine PB12 and PB23 membranes contained a total of 200 and 162 polymer chains, respectively. All BCP membranes were solvated using the solvate plugin in VMD. Water molecules placed within the PB layer were removed, whereas those that were placed in the PEO layer were kept. We conducted equilibration simulations of BCP membranes in three steps. In step 1, membranes were equilibrated in an NVT ensemble for 0.25 ns using a 1 fs time step after minimizing the system for 4000 steps. All atoms were fixed during the first phase of simulation except for the PB chains. In step 2, the time step was increased to 2 fs and the system was restarted after a brief minimization in an NPT equilibration using a constant area for 0.50 ns. During the second step, we applied external forces using a tcl-script to keep water molecules out of the PB layer. In step 3, all constraints on water molecules were removed and the system was restarted for long timescale MD simulations (Supplementary Table 2). We report equilibrated snapshots of pristine BCP membranes in Supplementary Fig. 12. We recorded trajectory data every 20 ps.

The POPC membrane built using the membrane builder plugin in VMD was first aligned by moving its center of mass to the origin. The PAP channel was then added to the system and embedded in the membrane by aligning its center of mass to the origin as well. Any overlapping lipid molecules within 1.5 Å of PAP were removed. After removing the unwanted lipids, the system was solvated using VMD’s solvate plugin. Any water molecules placed within the lipid bilayer were removed from the system. We carried out equilibration simulations of PAP embedded in the POPC membrane in three sequential steps. In the first step all atoms were kept fixed except those in the lipid tails. The system was then briefly minimized and equilibrated in an NVT ensemble for 0.25 ns using a 1 fs time step. The restart files from step 1 were then used to initiate step 2. During step 2, the system was equilibrated in an NPT ensemble for 0.5 ns using a 2 fs time step. Previous constraints placed on lipid and water molecules were released. During this simulation, the PAP channel was again constrained and a tcl-forces script was used to keep water out of the lipid membrane. Restart files from this simulation were used to initiate the final step. In step 3, we released all constraints and carried out long timescale equilibration simulations in an NPT ensemble using a 2 fs time step. Specifically, we carried out four independent trajectories of varying lengths (Supplementary Table 2). We recorded trajectory data every 20 ps. For PAP embedded in PB_12_-PEO_9_ and PB_23_-PEO_16_ membranes, we chose equilibrated configurations from pristine membrane simulations for inserting PAP. A similar procedure to the POPC membrane was followed for the alignment of PAP in these membranes, where unwanted polymer chains were deleted. We carried out three PAP-embedded MD simulations for each BCP membrane (Supplementary Table 2).

We computed diffusion coefficients using the well-known Einstein’s relation. The coordinates of all atoms in trajectories were first unwrapped. We divided trajectories into 20 ns fragments and realigned them to initial frames for computing the MSD averaged across all 20 ns fragments. Three different diffusivity coefficients were measured for diffusion of a single PAP channel in the membrane: the overall diffusivity coefficient, the lateral diffusivity coefficient (in the plane of the membrane), and the vertical diffusivity coefficient (in the *z*-direction normal to the membranes surface). The permeability of the PAP channel was measured using a collective diffusion model first proposed by Zhu et al.^41^.

To validate the selectivity of PAP channels, we performed a series of steered MD simulations on two selected dye molecules^75^. We chose MO (328 Da) and RB (1018 Da), based on the selectivity experiments of PAP with MO able to transport through the channel and RB rejected. We tested this conjecture by pulling each molecule through the central ring of PAP for 10 times (Supplementary Fig. 28C, and 28D). Structures and parameters for the dyes were generated using the CHARMM-GUI Ligand Reader and Modeler^76^. Systems were prepared by taking the initial POPC simulation structures and adding a single dye molecule displaced 10 Å from the central ring of PAP. Harmonic constraints with a force constant of 2 kcal mol^−1^ were placed on the dye and the central ring of PAP to keep them in place while the rest of the system was equilibrated in the NPT ensemble at 303 K and 1 atm for 25 ns. Pulling runs were performed at a rate of 10 Å ns^−1^ with a spring constant of 1000 kcal mol^−1^. Harmonic constraints with a force constant of 2 kcal mol^−1^ were kept on the central ring of PAP to hold it in place. The simulation runs were averaged together and then integrated to construct the potential of mean force for each molecule (Supplementary Fig. 28E). These free energy values could explain the selectivity of PAP channels to different dye molecules.

### Fabrication and characterization of composite membranes

PAP[5]/PB-PEO 2D sheets were made via the slow dialysis method as mentioned above. Carboxylic PB12 copolymers were used so that the final 2D sheets were fully carboxylic terminated. The molar channel to polymer ratio was control within 0.3–0.5 to so that the majority of the PAP[5]/PB-PEO aggregates were in the form of giant collapsed vesicles and 2D sheets. A total of 50 nm track-etched PC membranes and 30 nm PES membranes were used as substrates. They were first treated in a UV/Ozone cleaner for 30 s in order to ionize the surface (the shiny side) to obtain a negatively charged surface. The cleaned membranes, of 2.5 cm diameter, were placed onto a stainless steel mesh and assembled into a stirred cell (Model 8010, Millipore Corp., MA). The membranes were covered with 1 ml polyelectrolyte solution containing 40 mM PEI (60,000 Da), 35 mM CaCl_2_, and 0.5 M NaCl at pH of 5.5^48^. After incubation for 15 min, the solution was discarded, and the membranes were rinsed with DI water and replaced with 10 μl 2D sheets suspension diluted to 1 ml using the same buffer at pH of 8. The suspension was then filtered through the membranes after another 15 min. Thus, one layer of PEI and 2D sheets was physically deposited onto the substrates. After a desired number of layers was immobilized, the membranes were incubated overnight with 1 mg ml^−1^ 1-Ethyl-3-(3-dimethylaminopropyl)carbodiimide, 1 mg ml^−1^ N-Hydroxysuccinimide, and 10 mM potassium phosphate at pH of 7. The incubation chemically cross-linked the amine groups from PEI and the carboxylic groups from the 2D sheets. We also prepared the membranes only deposited with PEI as the PEI control membranes.

Carboxylic PB12 BCPs were used for the BCP control membrane fabrication. Free-standing PB-PEO 2D sheets were prepared by a solvent cast method^65^. In brief, 3% poly(vinyl alcohol) (PVA) solution in water (w/v%) was spin coated on the top of UV/ozone cleaned silicon substrate at 2000 rpm. In toal, 1% PB-PEO solution in tetrahydrofuran (THF) (w/v%) was prepared by stirring at 600 rpm for overnight, and this solution was drop-casted on the top of PVA layer and the casted PB-PEO layer was annealed during the slow evaporation of THF for overnight. After solvent evaporation, the silicon substrate was gently immersed into water at a tilted angle, allowing the annealed polymer film floating onto the water after dissolution of PVA. The supernatant solution, which is containing free-standing PB-PEO 2D sheets, were used for the block copolymer control membrane fabrication, using the aforementioned layer-by-layer approach.

The modified membranes were characterized on a scanning electron microscope at 5 keV. The *d*-spacing between PAP[5] based 2D sheets of dry and wet membranes were determined by X-ray diffraction (XRD)^66^. Flux was measured at 50 psi, 60 psi, and 70 psi, respectively, after the membranes were compressed at 50 psi for 30 min. A series of small molecular weight dyes with different charges were selected for rejection tests (Supplementary Table 4). Each dye was dissolved in DI water at a concentration of 35 μM (except for fluorescent dextran, the concentration was 6.7 μM) and loaded into a feed tank that was connected to the 10 ml stirred cell. The stirring speed was 300 rpm. The concentrations of different dye molecules were measured on a UV-Vis spectrophotometer. The initial 10 ml filtrate was discarded because of the adsorption by polyelectrolyte PEI. After 10 ml filtration, we observed that the dye concentrations in permeate became constant. We also observed significant dye rejections of the PAP[5] based membranes, which indicated a severe concentration polarization in the stirred cell, despite of the 300 rpm stirring employed. The concentrations of feed should be replaced with the dye concentrations on the membranes. The actual rejection was corrected using the stagnant film model^77,78^. We use a simple logistic function (a type of sigmoid function)^79^ to characterize the S-shaped curve of the molecular weight cutoff data and its derivative is a continuous probability distribution, which is a probability density function can be used to determine the pore size distribution.

### Data availability

The raw data that support the findings of this study are available in Penn State’s ScholarSphere repository with the identifier(s) [10.18113/S1KW61].

## Electronic supplementary material

Supplementary Information

Description of Additional Supplementary Files

Supplementary Movie 1

Supplementary Movie 2
