# Publisher Correction: Achieving high permeability and enhanced selectivity for Angstrom-scale separations using artificial water channel membranes

**DOI:** 10.1038/s41467-018-05447-3

**Published:** 2018-08-14

**Authors:** Yue-xiao Shen, Woochul Song, D. Ryan Barden, Tingwei Ren, Chao Lang, Hasin Feroz, Codey B. Henderson, Patrick O. Saboe, Daniel Tsai, Hengjing Yan, Peter J. Butler, Guillermo C. Bazan, William A. Phillip, Robert J. Hickey, Paul S. Cremer, Harish Vashisth, Manish Kumar

**Affiliations:** 1 Department of Chemical Engineering, The Pennsylvania State University, University Park, PA 16802 USA; 2 Department of Chemical Engineering, University of New Hampshire, Durham, NH 03824 USA; 3 Department of Chemistry, The Pennsylvania State University, University Park, PA 16802 USA; 4 Center for Polymers and Organic Solids, University of California at Santa Barbara, Santa Barbara, CA 93106 USA; 5 Department of Biomedical Engineering, The Pennsylvania State University, University Park, PA 16802 USA; 6 Department of Chemical and Biomolecular Engineering, University of Notre Dame, Notre Dame, IN 46556 USA; 7 Department of Material Science and Engineering, The Pennsylvania State University, University Park, PA 16802 USA; 8 Department of Civil and Environmental Engineering, The Pennsylvania State University, University Park, PA 16802 USA; 9 Present Address: Department of Chemistry, University of California, Berkeley, CA 94720 USA

Correction to: *Nature Communications*; 10.1038/s41467-018-04604-y; published online 12 June 2018

The original version of this Article contained an error in the spelling of the author Woochul Song, which was incorrectly given as Woochul C. Song. This has been corrected in both the PDF and HTML versions of the Article.

