# Supplementary material for: Achieving high permeability and enhanced selectivity for Angstrom-scale separations using artificial water channel membranes
Source: Nat Commun. 2018 Jun 12;9:2294. doi: 10.1038/s41467-018-04604-y (PMC5997692; doi:10.1038/s41467-018-04604-y)
Supplement: Supplementary file 1 — Supplementary Information [file 41467_2018_4604_MOESM1_ESM.pdf]

Supplementary Information

Achieving high permeability and enhanced selectivity for Angstrom-scale separations using artificial water channel membranes

Shen, Song *et al.*

Supplementary Methods

Chemicals

Peptide-appended pillar[5]arene (PAP) channels were synthesized using a previously published method[1]. Lipids were purchased from Avanti Polar Lipids (Alabaster, AL) and used without further purification. Poly(butadiene)-*b*-poly(ethylene oxide) (denoted as PB-PEO) diblock copolymers were synthesized using a previously described method[2] with slight modifications or purchased from Polymer Source. Briefly, anionic polymerization of 1,2-polybutadiene was performed in tetrahydrofuran using sec-butyl lithium as initiator and endcapped with ethylene oxide. The resulting monohydroxyl-terminated polybutadiene precursor was converted to the potassium alkoxide by titration with potassium naphthalenide. PEO growth was accomplished on the macroinitiator and the addition of slightly acidic methanol terminated the polymerization and yielded the hydroxyl-terminated PB-PEO copolymers. The block compositions were first estimated using ^{1}H NMR spectroscopy, and further confirmed by gel permeation chromatography. Carboxylic acid terminated PB-PEO (PB12) was synthesized by using succinic anhydride as a terminating agent. Supplementary Table 1 shows the detailed information of the selected PB-PEO copolymers with different polymer lengths.

Preparation of polymer and lipid vesicles

Polymersomes and liposomes were prepared by using the film rehydration method[3] with added channels. PAP channels (between 0 and 0.22 mg) were added to 8 mg block copolymers (BCPs) or 6 mg of a 4:1 (mol/mol) phosphatidylcholine/phosphatidylserine (PC/PS) mixture in $CHCl_3$. The mixture was gently dried on a rotary evaporator followed at high vacuum to remove all solvent. The formed film was rehydrated with 1 ml buffer containing 10 mM Hepes, 100 mM PEG600 and 0.01% (w/v) NaN_3 at pH of 7. The suspension was extruded through 0.2 μm track-etched membranes (Whatman, UK) at least 10 times to obtain monodisperse and unilamellar vesicles, after incubation on a stir plate at 4°C overnight. The size of the vesicles was measured using dynamic light scattering method on a Zetasizer Nano instrument (Malvern Instruments Ltd., UK).

Water permeability tests

The water permeability tests of polymer and lipid vesicles were conducted on an SF-300X stopped-flow instrument (KinTek Corp., PA) at 10°C, where vesicles were rapidly exposed to a hypotonic osmolyte (10 mM Hepes and 0.01% (w/v) NaN_3 at pH of 7), causing them to swell because of the inwardly osmotic gradient (lacking the 100 mM PEG600 in the buffer used to form the vesicles). The resulting changes in vesicle size caused the light scattering to increase at a wavelength of 600 nm and an angle of 90°[4]. The light scattering signal was fit in the form of the sum of two exponential equations. The osmotic permeability (P_f) was calculated using the following formula:[5]

$$P_f = \frac{k}{(S/V_0)\times V_W \times \Delta_{\mathrm{osm}}} \quad (1)$$

where k is the larger exponential coefficient describing the initial change in light scattering; S and V_0 are the initial surface area and volume of the vesicles; V_W is the molar volume of water, and Δ_{osm} is the osmolarity difference. Considering the non-ideality of the solution, the osmolarity difference values were measured on a freezing point based osmometer (Model 3300, Advanced Instruments, Inc., MA).

Labeling PAP channels

PAP channels were labeled with a rhodamine based fluorophore (5-(and-6)-((N-(5-aminopentyl)amino)carbonyl)tetramethylrhodamine) (Invitrogen, CA) using a cross-linker dicyclohexylcarbodiimide (DCC) (Thermo Fisher Scientific Inc., IL)[6]. The labeled channels were used to count the channel number per vesicle in fluorescence correlation spectroscopy (FCS) experiments, visually confirm the channel insertion into polymersomes using giant unilamellar vesicles (GUVs) technique and study the diffusion properties of PAP channels in lipid or polymer bilayers in fluorescence recovery after photobleaching (FRAP) experiments.

Fluorescence correlation spectroscopy (FCS)

The number of PAP channels per vesicle was measured based on a FCS technique[7,8]. Fluorescently labeled channels were incorporated into polymer of lipid vesicles using the film rehydration method[3] with the rehydration buffer (10 mM Hepes, 100 mM NaCl and 0.01% (w/v) NaN_3 at pH of 7). The vesicles were dialyzed, extruded and subjected to size-exclusion chromatography to remove residual free dye. The fluorescence intensity $F(t)$ within a small confocal volume was monitored using a time-resolved single-photon counting module (Becker-Hickl GmbH, Germany) to obtain the autocorrelation function $G(\tau)$ based on the fluorescence fluctuation $\delta F(t)$, t (time) and τ (time lag):

$$G(\tau) = \frac{\langle \delta F(t) \rangle \langle \delta F(t+\tau) \rangle}{\langle F(t) \rangle^2} \quad (2)$$

$$G(\tau) = \frac{1}{N} \sum_{i=1}^{M} f_i \left[\frac{1}{1+\tau/\tau_{D_i}} \right] \left[\frac{1}{1+(r/z)^2 (\tau/\tau_{D_i})} \right]^{1/2} \quad (3)$$

The specific expression of $G(\tau)$ is related to the geometry of the confocal volume (r and z are the radius and half height of the confocal volume), the 2D lateral diffusion time (τ_{Di}) of the fluorescent species i, and the fraction (f_i) of fluorescent species i. When τ=0, the number of the independent fluorescent molecules in the confocal volume (N) is the inverse of $G(0)$. The number of labeled channels per vesicle can be calculated as the ratio of the number of particles in the confocal volume before and after detergent solubilization of the vesicles (Supplementary Fig. 2). Because the labeled PAP channels showed significantly different diffusion times compared to the free dye[6], the autocorrelation curves were fit to the two species model. The theoretical insertion number was calculated in the following way: With a PB23 vesicle diameter of ~57 nm and assuming a bilayer thickness of 6 nm (Supplementary Table 1), the sum of outer and inner surface areas is $\pi/4\times d^2+\pi/4\times(d-5)^2$ =74,000 nm^2. The average cross-sectional areas of the PB23 and PAP channel are 0.88 nm^2 (Supplementary Fig. 9B) and 1.5 nm^2,[9] respectively. Assuming 100% of the channels are inserted, with an mCPR of 0.005, for example, the theoretical

insertion number of the channel is 210 per vesicle. The inserted channel number in PB12 and PB33 vesicles were obtained from Supplementary Fig. 7.

Giant unilamellar vesicles (GUVs) and confocal imaging

GUVs were constructed based on a cross-linked dextran (ethylene glycol) hydrogel protocol by Kros and coworkers[10]. Glass slides were first cleaned in the 1:1 (v/v) HCl/methanol mixture for 1 hour, rinsed with water, air-dried and left overnight in a glove box to exclude atmospheric moisture[11]. The dried slides were thiol-functionalized by being soaked in 2% (v/v) 3-mercaptopropyl trimethoxysilane in toluene for 1 hour and rinsed thrice in toluene in the glove box. After the reaction was complete, the slides were taken out of the glove box, rinsed with water and stored before further use. 420 µl of the 2 mM solution, 1:1 (mol/mol) maleimide-dextran and thiol conjugated PEG (Cellendes GmbH, Reutlingen, Germany) mixture, were immediately pipetted onto the glass slide after mixing. The coated hydrogel was covered with a piece of Parafilm M, yielding a uniform layer on the glass. A homogenous polymeric film was formed after 30–45 minutes at 40 °C. 10 µl of 1 mg·ml^{-1} BCPs in $CHCl_3$ (with labeled PAP channels) was pipetted on to the hydrogel film and dried for 30 minutes under vacuum at room temperature. The dried BCP film, confined in a GUV well growth chamber formed by placing an X-profiled O-Ring on top of the hydrogel and sealing with high vacuum silicon grease, was hydrated with 300 µl water and left at 40 °C for 1–2 hours to form free-floating vesicles. The image was taken on Leica TCS SP5 confocal microscope (LSCM, Leica Microsystems)

DSSN+ experiments

5 % (mol/mol) DSSN+ was added into lipid and polymer vesicles. After the final vesicle concentrations were diluted to 3 mg·ml^{-1}, these vesicles were incubated at 4 °C overnight. The UV-vis absorbance and emission spectra were scanned on a micro-plate reader (SpectraMax® i3X, Molecular Devices, CA)

Self-assembly of PAP channel/polymer aggregates

PAP channel/lipid aggregates were produced by a slow dialysis procedure[12]. First, 60 µg PB12 or carboxylic PB12 copolymers and PAP channels (0 to 320 µg) were dissolved in $CHCl_3$ and mixed at different mCPRs. After evaporating $CHCl_3$, the film was dissolved in 60 µl dialysis buffer (10 mM Hepes, 100 mM NaCl and 0.01% (w/v) NaN_3 at pH of 7) initially containing 4% (w/v) *n*-octyl-β-D-glucoside (OG). The PAP channel/polymer mixture was transferred into dialysis buttons (Hampton Research, CA) with a 12-kDa cut-off membrane, where the final polymer concentration was 1 mg·ml^{-1}. The detergent concentration (initially 4%, w/v) was gradually lowered by doubling the dialysis buffer volume with detergent-free buffer every 8 h until the OG concentration in the dialysis buffer reached 0.25% (w/v). The dialysis buffer was then replaced with detergent-free buffer three times every 8 h.

Transmission electron microscopy (TEM)

The polymersomes and PAP channel/BCPs aggregates were adsorbed on glow-discharged carbon-coated

TEM grids (Ted Pella Inc., CA), blotted, washed and stained with 0.75% uranyl formate. Conventional TEM, energy filtered TEM and scanning TEM with energy dispersive spectroscopy (EDS) map were performed on a Tecnai G2 Spirit BioTwin microscope (FEI) and a Tecnai G2 20 X-Twin microscope (FEI), and a Talos F200X microscope (FEI), respectively. For Cryo-TEM, 5 μL polymersome solution was loaded onto QUANTIFOIL® holey carbon TEM grids (Ted Pella Inc., CA). The excess liquid was blotted and the grids were vitrified in liquid ethane using a Vitrobot (FEI). The images were captured in low-dose mode a Tecnai G2 Spirit BioTwin microscope (FEI).

Fluorescence recovery after photobleaching (FRAP)

FRAP experiments[13] were conducted to investigate the diffusion behavior of PAP channels in BCP bilayers at room temperature. The PB-PEO polymersomes with labeled PAP incorporated were made using the aforementioned film rehydration method. Additionally, 0.5 % (mol/mol) 1-oleoyl-2-[12-[(7-nitro-2-1,3-benzoxadiazol-4-yl)amino]dodecanoyl]-*sn*-glycero-3 phosphoethanolamine (NBD-PE) was added into the vesicles during the film rehydration as a reference. The supported bilayers were formed after the vesicles were ruptured on a cleaned glass coverslip in a PDMS well, with the addition (1:1) of a buffer containing 33mM $CaCl_2$ and 10mM Tris at pH of 7.4. After a fusion time of 30-60 min, these bilayers were fixed and rinsed with water, a small section of the bilayers were removed by scratching the surface of the glass coverslip with a tweezer. The FRAP experiments were conducted on a Nikon Eclipse TE-2000-U inverted microscope through a 10× objective lens. The 463 nm and 532 nm lasers (Stabilite 2018, Spectra Physics) were used to bleach NBD-PE and fluorescent labeled PAP channels in the bilayers, respectively. The diameter of the spot was 20 μm. The fluorescence intensity in the selected area was monitored at every 30 s at the beginning, then reduced to one image per 15-30 min. The fluorescence recovery curves were used to determine the mobile fraction and diffusion coefficients of PAP channels in the bilayers. The fluorescence intensity of the bleached spot at any time was divided by the fluorescence intensity of an unbleached spot in order to correct for differences in light intensity and photobleaching. The percentage of the normalized fluorescence recovery $f(t)$ was calculated using the following equation, where I_t is the normalized intensity of the spot at time t, I_0 is the normalized intensity of the spot immediately after bleaching and I_i is the initial averaged intensity,

$$f(t) = \frac{I_t - I_0}{I_i - I_0} \quad (4)$$

The recovery as a function of time was fit to an exponential equation in order to obtain the mobile fraction of the bilayer (A) and the time of half recovery $\tau_{1/2}$,

$$\tau_{1/2} = \frac{1}{\tau} \times ln2 \quad (5)$$

$$f(t) = A(1 - e^{-\tau t}) \quad (6)$$

The lateral diffusion coefficient (D) is calculated by employing the following formula[13], where ω is the half-width of the Gaussian laser beam (20 μm).

$$D = \frac{0.88\times\omega^2}{4\times\tau_{1/2}} \quad (7)$$

Estimation of the hydrophobic core thickness of the BCP polymersomes and the cross-sectional area of per polymer chain. The hydrophobic core thickness (*d*) of the BCPs bilayer membrane has a logarithmic relationship with the hydrophobic molecular weight (M_h) with a scaling exponent of a, if the BCPs have the similar hydrophobic volume fraction[14]. By assuming incompressibility, the cross-sectional area of per polymer chain (*A*) should also scale with an exponent of b[15].

$$d \sim M_h^a \quad (8)$$

$$A \sim M_h^b \quad (9)$$

Using the published PB-PEO and its reduced polymer poly(ethylethylene)-*b*-poly(ethylene oxide) (PEE-PEO) data[14,16,17], we obtained the scale components a and b with the hydrophobic molecular weight of the polymer and estimated the hydrophobic core thickness and the cross-sectional area of per polymer chain of PB12, PB23 and PB33 BCPs (Supplementary Table 1 and Supplementary Fig. 9).

Simulation methods

Simulation Setup, Software, and Force-field Parameters

We conducted all molecular dynamics (MD) simulations using NAMD[18] as our simulation engine, and used VMD[19] for visualization and analyses. The temperature and pressure in simulations were controlled at 303 K and 1 atm, using a Langevin thermostat and a Nose-Hoover barostat, respectively. We used the latest version of CHARMM force-field to simulate water, lipids, and polyethylene oxide[20]. The parameters used to simulate the PAP channel were identical to the previous work.[6] The necessary polymer chains for BCP membranes were created by first generating monomers using the molefacture plugin in VMD. Molefacture generates a pdb/psf pair and a topology file for each monomer. We then patched monomers together using VMD's psfgen tool to form BCP chains of desired lengths. An existing patch was used to generate PEO chains, but new patches were built to join PB polymer blocks as well as to combine PB blocks with PEO blocks. All missing parameters for PB blocks and PB-PEO linkage were generated iteratively using the Force Field Toolkit (FFTk) plugin in VMD in combination with Gaussian quantum chemistry software. Specifically, to create PB blocks of any length, we parameterized a PB chain containing two monomers for which Lennard-Jones non-bonded parameters were assigned from existing parameter files in the CHARMM force-field. Gaussian was then used to optimize the geometry and to assign charges on all atoms. The missing parameters were generated and optimized using FFTk plugin in VMD and Gaussian software.

Construction of pristine membranes

We used the membrane builder plugin in VMD to generate the POPC membrane. The POPC membrane used in our simulations was 70 Å×70 Å in size. However, BCP membranes were generated by first constructing a single polymer chain of desired length for both PB and PEO blocks. The two independent

chains were then connected using a patch, the parameters of which were optimized as described above. Each connected PB-PEO chain was then replicated and placed in a regular 10×10 grid pattern over an area of 80 Å×80 Å (PB12) and 9×9 grid pattern over an area of 80 Å×80 Å (PB23). All BCP membranes were arranged in a di-block configuration (Supplementary Fig. 12) prior to equilibration. Pristine PB12 and PB23 membranes contained a total of 200 and 162 polymer chains, respectively. All BCP membranes were solvated using the solvate plugin in VMD. Water molecules placed within the PB-layer were removed, while those that were placed in the PEO layer were kept. We conducted equilibration simulations of BCP membranes in three steps. In step 1, membranes were equilibrated in an NVT ensemble for 0.25 ns using a 1 fs time step after minimizing the system for 4000 steps. All atoms were fixed during the first phase of simulation except for the PB chains. In step 2, the time step was increased to 2 fs and the system was restarted after a brief minimization in an NPT equilibration using a constant area for 0.50 ns. During the second step, we applied external forces using a tcl-script to keep water molecules out of the PB layer. In step 3, all constraints on water molecules were removed and the system was restarted for long timescale MD simulations (Supplementary Table 2). We report equilibrated snapshots of pristine BCP membranes in Supplementary Fig. 12. We recorded trajectory data every 20 ps.

PAP embedded in POPC and BCP Membranes

The POPC membrane built using the membrane builder plugin in VMD was first aligned by moving its center of mass to the origin. The PAP channel was then added to the system and embedded in the membrane by aligning its center of mass to the origin as well. Any overlapping lipid molecules within 1.5 Å of PAP were removed. After removing the unwanted lipids, the system was solvated using VMD's solvate plugin. Any water molecules placed within the lipid bilayer were removed from the system. We carried out equilibration simulations of PAP embedded in the POPC membrane in three sequential steps. In the first step all atoms were kept fixed except those in the lipid tails. The system was then briefly minimized and equilibrated in an NVT ensemble for 0.25 ns using a 1 fs time step. The restart files from step 1 were then used to initiate step 2. During step 2, the system was equilibrated in an NPT ensemble for 0.5 ns using a 2 fs time step. Previous constraints placed on lipid and water molecules were released. During this simulation, the PAP channel was again constrained and a tcl-forces script was used to keep water out of the lipid membrane. Restart files from this simulation were used to initiate the final step. In step 3, we released all constraints and carried out long timescale equilibration simulations in an NPT ensemble using a 2 fs time step. Specifically, we carried out 4 independent trajectories of varying lengths (Supplementary Table 2). We recorded trajectory data every 20 ps. For PAP embedded in PB_{12}-PEO_9 and PB_{23}-PEO_{16} membranes, we chose equilibrated configurations from pristine membrane simulations for inserting PAP. A similar procedure to the POPC membrane was followed for the alignment of PAP in these membranes, where unwanted polymer chains were deleted. We carried out three PAP embedded MD simulations for each BCP membrane (Supplementary Table 2).

Diffusivity and permeability Calculations

We computed diffusion coefficients using the well-known Einstein's relation. The coordinates of all atoms in trajectories were first unwrapped. We divided trajectories into 20 ns fragments and realigned them to initial frames for computing the mean-square-displacement (MSD) averaged across all 20 ns fragments. Three different diffusivity coefficients were measured for diffusion of a single PAP channel in the membrane: the overall diffusivity coefficient, the lateral diffusivity coefficient (in the plane of the membrane), and the vertical diffusivity coefficient (in the z-direction normal to the membranes surface). The permeability of the PAP channel was measured using a collective diffusion model first proposed by Zhu *et al*[21]. This method has been used previously to measure the permeability of the PAP channel in a lipid membrane[6]. The MSD of the collective displacement coordinate n was obtained from the embedded simulations in POPC, PB12 and PB23 membranes. The channel volume of interest was defined the same way as in the previous work[6] as a cylinder 6 Å in diameter, 8 Å in length, normal to the membrane surface, and aligned with the center of mass of the carbons of the dimethoxy-benzene-rings of the PAP channel. Using water in this defined volume, the collective displacement coordinate, $n(t)$, was calculated for each trajectory. The $n(t)$ trajectories were split into 1 ns segments and realigned such that $n(0) = 0$ and averages across all trajectory fragments were taken as the value of $\langle n^2 \rangle (t)$. This value was plotted to determine the permeability of the channel.

Selectivity validation

In order to validate the selectivity of PAP channels, we performed a series of steered molecular dynamics simulations on two selected dye molecules.[22] We chose methyl orange (MO, 328 Da) and rose bengal (RB, 1,018 Da), based on the selectivity experiments of PAP with MO able to transport through the channel and RB rejected. Upon a cursory analysis of each chemical structure, we assume that shape plays a key role in PAP's relative selectivity. MO has a linear shape ideal for passing through the central ring of PAP without any significant deformation to the molecular structure (Supplementary Fig. 28A). RB consists of multiple carbon rings arranged in a tetrahedral shape (Supplementary Fig. 28B). There is no way for this molecule to pass through the central ring without a significant deformation. We tested this conjecture by pulling each molecule through the central ring of PAP for 10 times (Supplementary Fig. 28C and 28D). Structures and parameters for the dyes were generated using the CHARMM-GUI Ligand Reader and Modeler[23]. Systems were prepared by taking the initial POPC simulation structures and adding a single dye molecule displaced 10 Å from the central ring. Harmonic constraints with a force constant of 2 $kcal \cdot mol^{-1}$ were placed on the dye and central ring of PAP to keep them in place while the rest of the system was equilibrated in the NPT ensemble at 303 K and 1 atm for 25 ns. Pulling runs were performed at a rate of 10 $Å \cdot ns^{-1}$ with a spring constant of 1000 $kcal \cdot mol^{-1}$. Harmonic constraints with a force constant of 2 $kcal \cdot mol^{-1}$ were kept on the central ring of PAP to hold it in place. The stiff spring constant was used to minimize drift of the dye and ensure each molecule was pulled through the ring. The simulation runs were averaged together and then integrated to construct the potential of mean force for each molecule (Supplementary Fig. 28E). This value could explain the selectivity of PAP channels to different dye molecules.

Calculations of membrane permeability from osmotic permeability

The membrane permeability (A_{BCP}) of the block copolymer membrane can be related to the osmotic permeability (P_f)[24],

$$A_{BCP}(\mu m \cdot s^{-1} \cdot bar^{-1}) = \frac{P_f V_W}{RT} \quad (10)$$

where V_W is the molar volume of water, R is the universal gas constant and T is the absolute temperature. P_f can be measured as described above using the stopped-flow light scattering test by mixing the polymersomes with osmolyte. This permeability is driven by the osmolyte concentration gradient (Δ_{osm}) across the block copolymer bilayer.

PAP channels in block copolymer membranes are deposited onto the porous substrate on a 1 μm×1 μm membrane. The packing density is ~4.2×10^5 μm^{-2}. The unit channel permeability is 1.8×10^{-14} $cm^3 \cdot s^{-1}$ (from simulation results). We also estimate the permeability of block copolymer is ~40 $\mu m \cdot s^{-1}$.

$$P_{channel\ membrane} = \frac{N_{channel} \times P_{channel} + (S_{Membrane} - S_{channel}) \times P_{BCP}}{S_{Membrane}}$$

$$= \frac{420{,}000 \times 1.8 \times 10^{-14} cm^3 \cdot s^{-1} + 1\ \mu m^2 \times 0.4 \times 40\ \mu m \cdot s^{-1}}{1\ \mu m \times 1\ \mu m} = 7{,}576\ \mu m \cdot s^{-1}$$

$$A_{channel\ membrane} = 7{,}576\ \mu m \cdot s^{-1} \times \frac{18\ cm^3 \cdot mol^{-1}}{83.1\ cm^3 \cdot bar \cdot\ mol^{-1} \cdot K^{-1} \times 283.15\ K}$$

$$= 5.80\ \mu m \cdot s^{-1} \cdot bar^{-1}$$

$$A_{channel\ membrane} = 5.80\ \mu m \cdot s^{-1} \cdot bar^{-1} = 5.80 \times \frac{\mu m}{s \cdot bar} \times \frac{1m/10^6\ \mu m}{1h/3{,}600\ s}$$

$$A_{channel\ membrane} = 0.021\ m \cdot h^{-1} \cdot bar^{-1} = 0.0021\ m \cdot h^{-1} \cdot bar^{-1} \times \frac{L/0.001m^3}{1m^2}$$

$$A_{channel\ membrane} = 21\ LMH \cdot bar^{-1}$$

Fabrication and characterization of PAP[5] 2D sheets based composite membranes

A modified layer-by-layer method was used to immobilize PAP[5]/PB-PEO 2D sheets on to support membranes. These sheets were made via the slow dialysis method as mentioned above. Carboxylic PB12 copolymers were used so that the final 2D sheets were fully carboxylic terminated. The molar channel to polymer ratio was control within 0.3 to 0.5 to so that the majority of the PAP[5]/PB-PEO aggregates were in the form of giant collapsed vesicles and 2D sheets. The final suspension of 2D sheets contained 0.5 $mg \cdot ml^{-1}$ PAP[5] channels. 50 nm track-etched polycarbonate (PC) membranes (Whatman, UK) and 30 nm polyethersulfone (PES) membranes (Sterlitech Corp., WA) were used as substrates. They were first treated in a UV/Ozone cleaner (Ossila Ltd., UK) for 30 sec in order to ionize the surface (the shiny side) to obtain a negatively charged surface. The cleaned membranes, of 2.5 cm diameter, were placed onto a stainless steel mesh (Transferra Nanosciences Inc., CA) and assembled into a stirred cell (Model 8010, Millipore Corp., MA). The membranes were covered with 1 ml polyelectrolyte solution containing 40 mM polyethyleneimine (PEI, 60,000 Da) (the concentration was based on the repeat unit molecular weight), 35 mM $CaCl_2$, and 0.5 M NaCl at pH of 5.5[25]. After incubation for 15

min, the solution was discarded, and the membranes were rinsed with DI water and replaced with 10 μl 2D sheets suspension diluted to 1 ml using the same buffer at pH of 8. The suspension was then filtered through the membranes after another 15 min. Thus, 1 layer of PEI and 2D sheets was physically deposited onto the substrates. After a desired number of layers was immobilized, the membranes were incubated overnight with 1 mg·ml^{-1} 1-Ethyl-3-(3-dimethylaminopropyl)carbodiimide (EDC), 1 mg·ml^{-1} N-Hydroxysuccinimide (NHS) and 10 mM potassium phosphate at pH of 7. The incubation chemically-crosslinked the amine groups from PEI and the carboxylic groups from the 2D sheets. We also prepared the membranes only deposited with PEI as control membranes.

Carboxylic PB12 (Supplementary Table 1) BCPs were used for the block copolymer control membrane fabrication. Free-standing PB-PEO 2D sheets were prepared by a solvent cast method as described elsewhere[26]. Briefly, 3% poly(vinyl alcohol) (PVA) solution in water (w/v%) was spin coated on the top of UV/ozone cleaned silicon substrate at 2,000 rpm. 1% PB-PEO solution in tetrahydrofuran (THF) (w/v%) was prepared by stirring at 600 rpm for over-night, and this solution was drop-casted on the top of PVA layer and the casted PB-PEO layer was annealed during the slow evaporation of THF for over-night. After solvent evaporation, the silicon substrate was gently immersed into water at a tilted angle, allowing the annealed polymer film floating onto the water after dissolution of PVA. The supernatant solution, which is containing free-standing PB-PEO 2D sheets, were used for the block copolymer control membrane fabrication, using the aforementioned layer-by-layer approach.

The modified membranes were dried, sputtered with 8 nm iridium (Leica EM ACE600, Germany) and then characterized on a scanning electron microscope (FEI Nova NanoSEM 630 FESEM, OR) at 5 keV. The *d*-spacing between PAP[5] based 2D sheets of dry and wet membranes were determined by X-ray diffraction (XRD). The experiments was performed with focused Cu-Ka radiation (λ=1.5406 Å) at a scan rate of 3° min^{-1}, with 2θ from 5° to 30° (PANalytical Empyrean, Netherlands). Considering the interference of water molecules on the membrane surface against XRD signals during the state conversion from wet to dry, a tailored protocol was used for wet membrane measurements[27]. Scanning was performed immediately after wetting the membrane, and repeated 4-5 times continuously until a characteristic diffraction peak of dry membrane appeared. The curve with the highest signal intensity before the membrane dried was selected as its lamellar structure. The *d*-spacing of the modified membranes was calculated using Bragg's law from the characteristic peak.

$$d = \frac{\lambda}{2sin\theta} \quad (11)$$

Flux was measured at 50 psi, 60 psi and 70 psi, respectively, after the membranes were compressed at 50 psi for 30 min. A series of small molecular weight dyes with different charges were selected for rejection tests. Each dye was dissolved in DI water at a concentration of 35 μM (except for fluorescent dextran, the concentration was 6.7 μM) and loaded into a feed tank (Model RC800, Millipore Corp., MA) that was connected to the 10 ml stirred cell. The stirring speed was 300 rpm. The concentrations of different dye molecules were measured on a UV-Vis spectrophotometer (NanoDrop 2000c, Thermo Scientific, MA). The initial 10 ml filtrate was discarded because of the adsorption by polyelectrolyte PEI. After 10 ml filtration, we observed that the dye concentrations in permeate became constant. The

apparent rejection was calculated as

$$R = 1 - \frac{c_{permeate}}{c_{feed}} \quad (12)$$

We observed significant dye rejections of the PAP[5] based membranes, which indicated a severe concentration polarization in the stirred cell, despite of the 300 rpm stirring employed. The concentrations of feed should be replaced with the dye concentrations on the membranes. The actual rejection should be calculated as the following equation and can be corrected using the stagnant film model[28,29],

$$R' = 1 - \frac{c_{permeate}}{c_{membrane}} = \frac{R}{R + e^{-\frac{J}{k}}(1 - R)} \quad (13)$$

Here mass transfer coefficient *'k'* for each dye can be determined from Colton-Smith empirical correlation[29-31],

$$Sh = 0.285\, Re^{0.567} Sc^{0.33}\ (8{\times}10^3 < Re \leq 3.2{\times}10^4)\ \ (14)$$

$$Sh = 0.0443\, Re^{0.746} Sc^{0.33}\ (3.2{\times}10^4 \leq Re < 8.2{\times}10^4)\ (15)$$

where Reynold number, $Re = \rho\omega r^2/\mu$, Schmidt number, $Sc = \mu/(\rho D)$ and Sherwood number, $Sh = kr/D$ with ρ, ω, μ, D and r being density, rotation speed (in radians per sec), viscosity, diffusivity and stirred cell effective radius (8.5 mm) respectively. J is the volumetric flux through the membrane. Diffusivity D can be calculated from Stokes-Einstein equation,

$$D = \frac{kT}{6\pi\mu a} \quad (16)$$

where k, T and a being Boltzmann constant, temperature and molecule radius respectively. Molecule radius a (nm) is estimated from the following equation[32],

$$a = 0.066 M^{1/3} \quad (17)$$

where M being the molecular weight. We assume each dye molecule has the simplest shape, a sphere, and the partial specific volume is similar to that of proteins.

We use a simple logistic function (a type of sigmoid function)[33] to characterize the S-shaped curve of the molecular weight cutoff data and its derivative is a continuous probability distribution, which is a probability density function can be used to determine the pore size distribution.

$$y = \frac{1}{1 + e^{-\gamma x + \beta}} \quad (18)$$

$$y' = \gamma \frac{e^{-\gamma x + \beta}}{(1 + e^{-\gamma x + \beta})^2} \quad (19)$$

The standard deviation (σ) of this pore size distribution is

$$\sigma = \frac{\pi}{\sqrt{3}\gamma} \quad (20)$$

Supplementary Tables

Supplementary Table 1. Poly(butadiene)-*b*-poly(ethylene oxide) (PB-PEO) diblock copolymers used in this study

Polymer ID	Polymer composition	Molecular weight ($g\cdot mol^{-1}$)	Hydrophilic volume fraction ($f_{hydrophilic}$)	Bilayer thickness (nm)[b]	Hydrophobic core thickness (nm)[c]	Area of per chain (nm^2)[d]
PB12[a]	PB_{12}-PEO_9	1,050	0.32	5.1±0.6	3.7	0.69
Carboxylic PB12	PB_{12}-PEO_8-COOH	1,000	0.29	5.1±0.6	3.7	0.69
PB23	PB_{23}-PEO_{16}	1,980	0.30	6.0±0.5	5.4	0.88
PB33	PB_{33}-PEO_{24}	2,932	0.31	7.4±0.6	6.8	1.0

[a] This polymer was purchased from Polymer Source.

[b] Estimated from Cryo-TEM (Supplementary Fig. 8).

[c, d] Estimated from the scaling of the data fitting from the published polymer data (Supplementary Fig. 9).

Supplementary Table 2. Details of all MD simulations in three types of membranes (POPC, PB12, and PB23)

Membrane	Type	Run	Size (Atoms)	Length (ns)
POPC	Embedded	Run 1	32,666	250
	Embedded	Run 2	32,666	250
	Embedded	Run 3	32,666	250
PB12	Equilibration	Run 1	81,421	100
	Embedded	Run 1	78,122	250
	Embedded	Run 2	78,122	250
	Embedded	Run 3	78,122	250
PB23	Equilibration	Run 1	124,125	100
	Embedded	Run 1	119,154	250
	Embedded	Run 2	119,154	250
	Embedded	Run 3	119,154	250

Supplementary Table 3. A summary of all MD simulations results of PAP[5] channels in three types of membranes (POPC, PB12, and PB23)

PAP[5] in different membranes	Permeability ($\times 10^{14}$ $cm^3 \cdot s^{-1}$)	Overall Diffusivity ($\mu m^2 \cdot s^{-1}$)	Lateral Diffusivity ($\mu m^2 \cdot s^{-1}$)	Vertical Diffusivity ($\mu m^2 \cdot s^{-1}$)	Backbone RMSD (Å)	Tilt angle (°)
POPC	5.4 ± 0.1	3.24 ± 0.01	4.37 ± 0.01	0.99 ± 0.01	3.9 ± 0.4	15.9 ± 7.0
PB12	1.80 ± 0.02	2.20 ± 0.01	3.08 ± 0.01	0.13 ± 0.01	2.7 ± 0.7	11.1 ± 5.2
PB23	2.48 ± 0.01	1.92 ± 0.01	2.76 ± 0.01	0.23 ± 0.01	2.4 ± 0.4	14.4 ± 4.2

Supplementary Table 4. A summary of the selected dye molecules used in the rejection tests of this study.

Dye (abbreviation)	MW (Da)	Charge	D ($m^2 \cdot s^{-1}$)	k ($m \cdot s^{-1}$)
Methyl orange (MO)	328	−	4.7×10^{-10}	1.6×10^{-5}
Crystal violet (CV)	408	+	4.4×10^{-10}	1.5×10^{-5}
Rhodamine B isothiocyanate	536	+	4.0×10^{-10}	1.4×10^{-5}
Acid fuchsin (AF)	585	−	3.9×10^{-10}	1.4×10^{-5}
Methyl blue (MB)	800	−	3.5×10^{-10}	1.3×10^{-5}
Rose bengal (RB)	1018	−	3.2×10^{-10}	1.2×10^{-5}
Fluorescent dextran (FD)	3000	−	2.3×10^{-10}	1.0×10^{-5}

Note: Diffusivity D can be calculated from Stokes-Einstein equation and mass transfer coefficient k can be estimated from Colton-Smith empirical correlation. The details can be found in the section 'Fabrication and characterization of PAP[5] 2D sheets based composite membranes'

Supplementary Figures

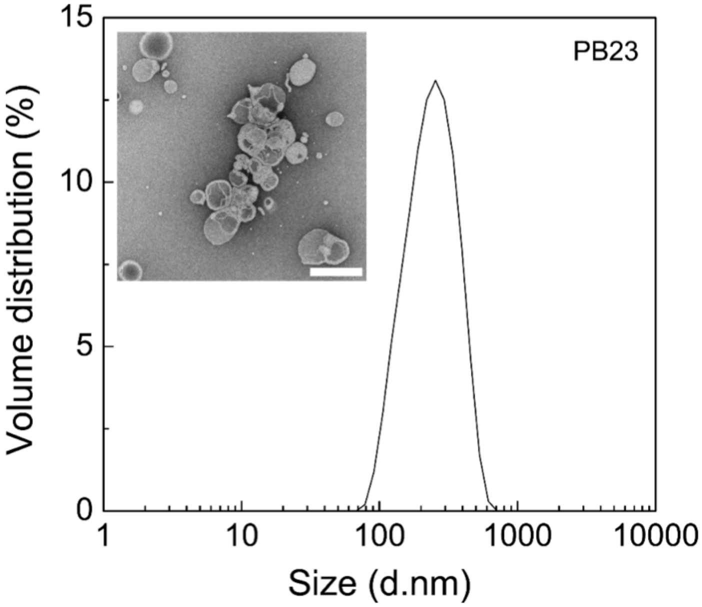

Supplementary Figure 1. Volumetric size distribution of the PB23 polymersomes at a molar channel to ratio of 0.005. The size distribution showed most of the film rehydration products were vesicles with a size of ~200-300 nm in diameter, as supported by the inset representative TEM image. The size distribution showed the majority of the self-assembled PB23 aggregates via the film rehydration method were vesicles. Scale bar, 500 nm.

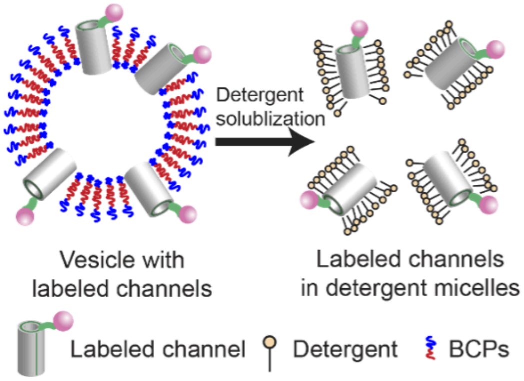

Supplementary Figure 2. FCS was used to determine the number of PAP channels per vesicle. First, FCS was used to analyze vesicles containing fluorescently labeled channels. The vesicles were then solubilized with detergent, releasing the channels into detergent micelles, and the solution was again analyzed by FCS.

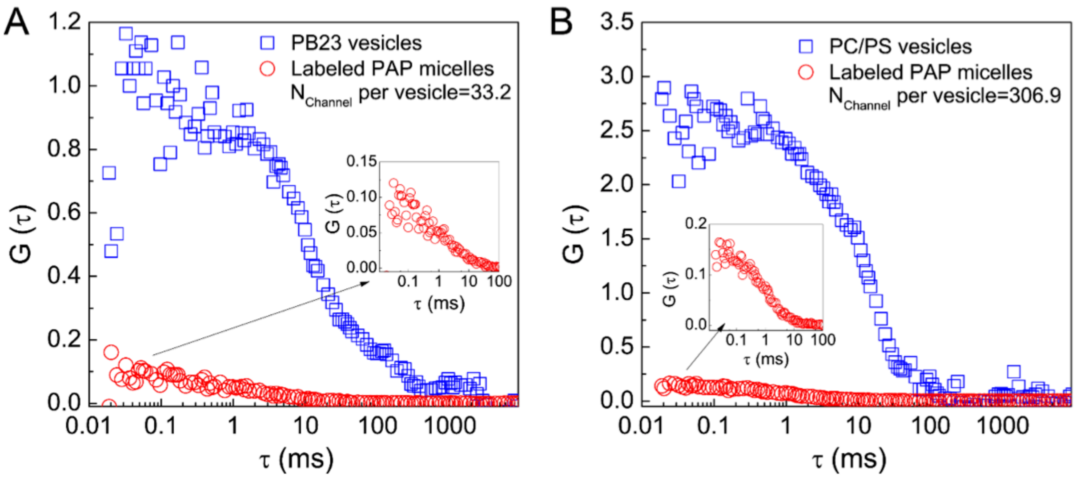

Supplementary Figure 3. Representative FCS autocorrelation curves for (A) PB23 vesicles with labeled PAP at an mCPR of 0.005, and (B) PC/PS liposomes with labeled PAP at an mCLR of 0.005 before and after solubilization with 2.5% OG. The high correlation function amplitude *G(0)* obtained for the vesicles indicates a low number of fluorescent vesicles in the confocal volume (N_{Vesicles}). After detergent solubilization, the number of free particles was released in micelles (N_{Micelles}). The number of channels per vesicle was then calculated as $N_{\mathrm{Micelles}}/N_{\mathrm{Vesicles}}$, after taking a dilution effect into account.

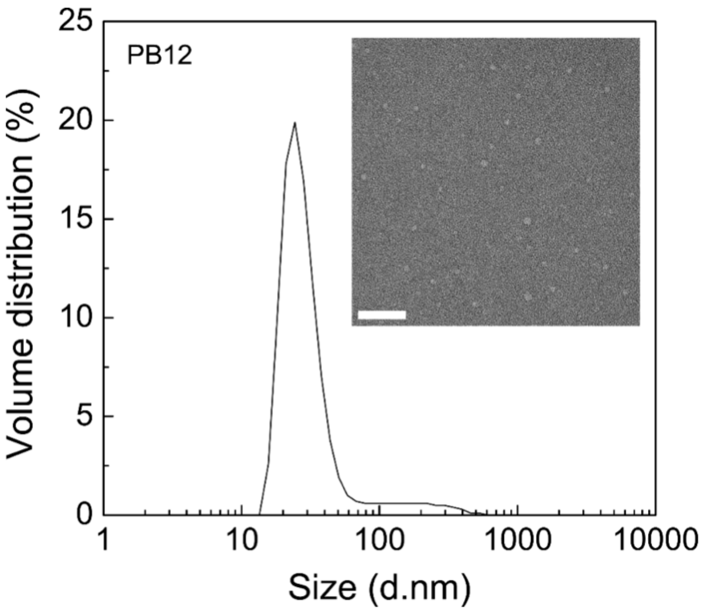

Supplementary Figure 4. Volumetric size distribution of the PB12 polymersomes at a molar channel to ratio of 0.005. The size distribution showed most of the film rehydration products were small particles with a size of ~20 nm in diameter, as supported by the inset representative TEM image. The size distribution showed the majority self-assembled PB12 aggregates via the film rehydration method were micelles. Scale bar, 100 nm.

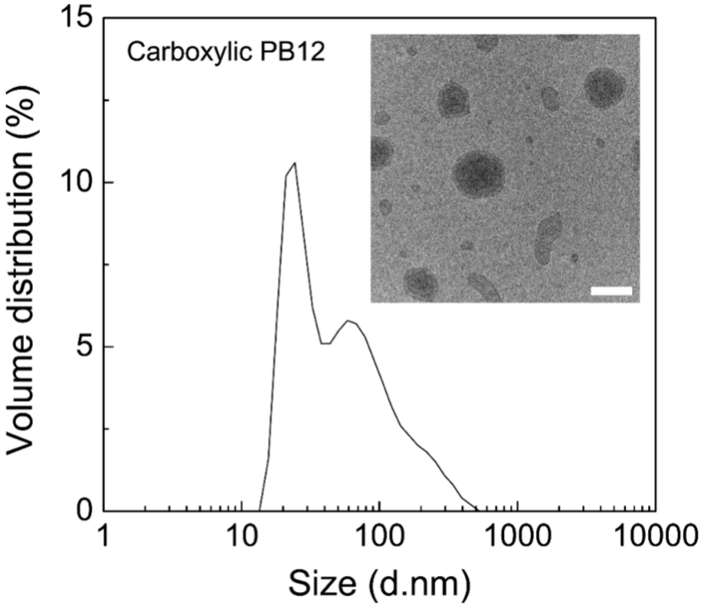

Supplementary Figure 5. Volumetric size distribution of the carboxylic PB12 polymersomes at a molar channel to ratio of 0.005. The size distribution showed the film rehydration products were a mixture of vesicles and small particles with a size of ~20 nm in diameter, as supported by the inset representative TEM image. The size distribution showed the majority of the self-assembled carboxylic PB12 aggregates via the film rehydration method were a mixture of micelles and vesicles. Scale bar, 500 nm.

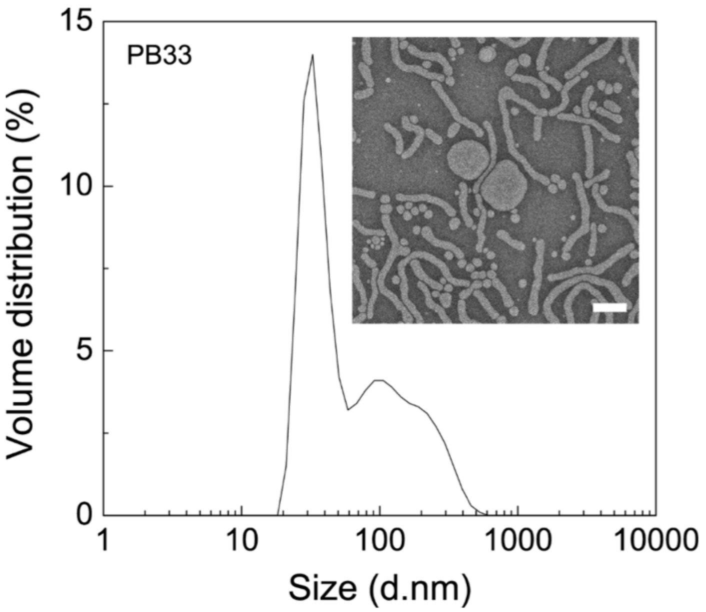

Supplementary Figure 6. Volumetric size distribution of the PB33 polymersomes at a molar channel to ratio of 0.005. The size distribution showed most of the film rehydration products were worm-like micelles aggregates with a size as supported by the inset representative TEM image. We still observed a small proportion of vesicles. The size distribution showed the majority of the self-assembled PB33 aggregates via the film rehydration method were worm-like micelles. Scale bar, 100 nm.

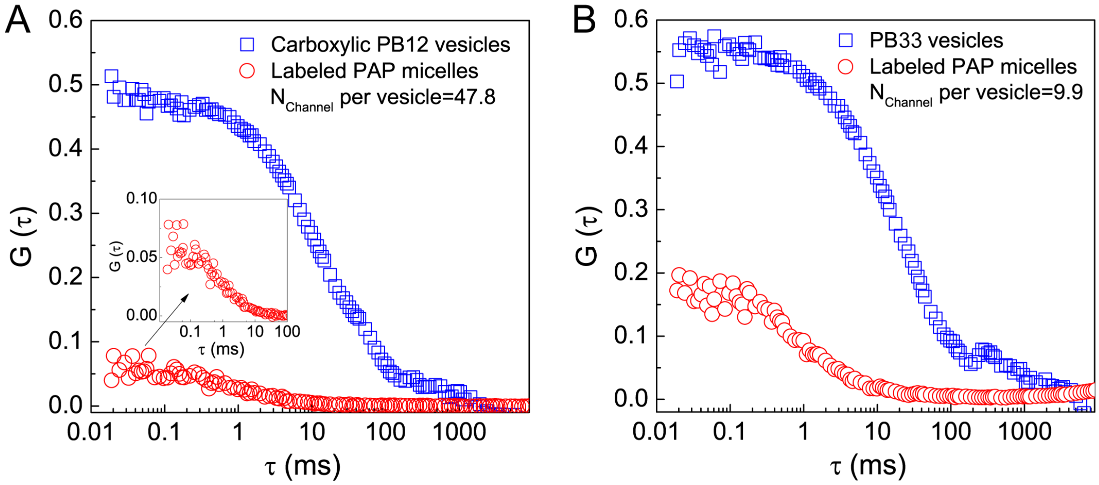

Supplementary Figure 7. Representative FCS autocorrelation curves for (A) carboxylic PB12 vesicles and (B) PB33 vesicles with labeled PAP at an mCLR of 0.005 before and after solubilization with 2.5% OG. The high correlation function amplitude *G(0)* obtained for the vesicles indicates a low number of fluorescent vesicles in the confocal volume (N_{Vesicles}). After detergent solubilization, the number of free particles was released in micelles (N_{Micelles}). The number of channels per vesicle was then calculated as $N_{\mathrm{Micelles}}/N_{\mathrm{Vesicles}}$, after taking a dilution effect into account.

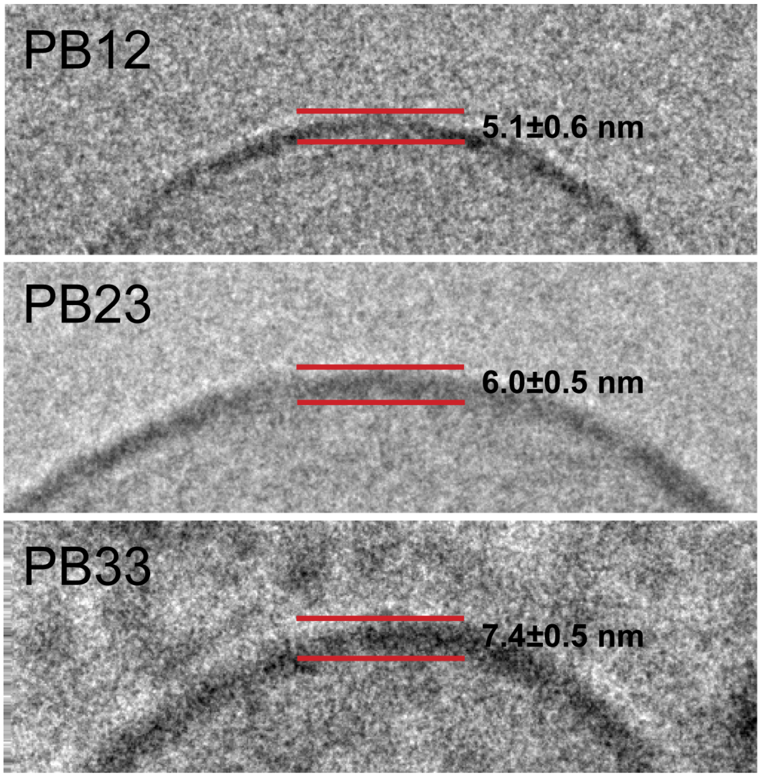

Supplementary Figure 8. Cryo-transmission electron microscopy showed the bilayer thickness of different PB-PEO polymersomes. Data shown are the average of 12 measurements with standard deviation.

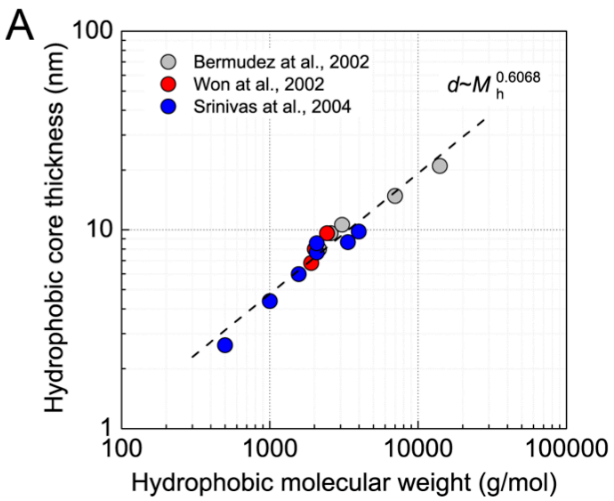

Supplementary Figure 9. Hydrophobic core thickness (d) of the bilayer membrane and cross-sectional area per chain (A) as the function of the hydrophobic molecular weight (M_h) for poly(butadiene)-*b*-poly(ethylene oxide) (PB-PEO) and its reduced poly(ethylethylene)-*b*-poly(ethylene oxide) (PEE-PEO) block copolymers.

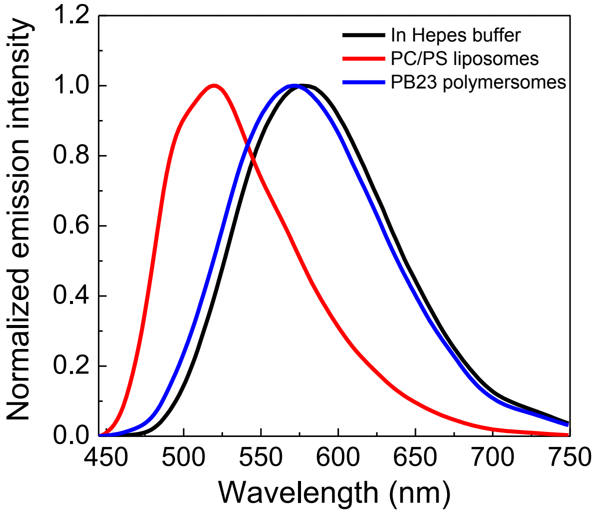

Supplementary Figure 10. The emission spectrum of DSSN+ in aqueous buffer containing 20 mM Hepes, 100 mM NaCl, 0.01 % (w/v) NaN_3, pH=7.4, and embedded within PC/PS lipid membranes, PB23 polymer membranes. DSSN+ displayed a blue shift in λ_{Em} due to the polarity difference in aqueous buffer and the hydrophobic membranes of the lipids and block copolymers.

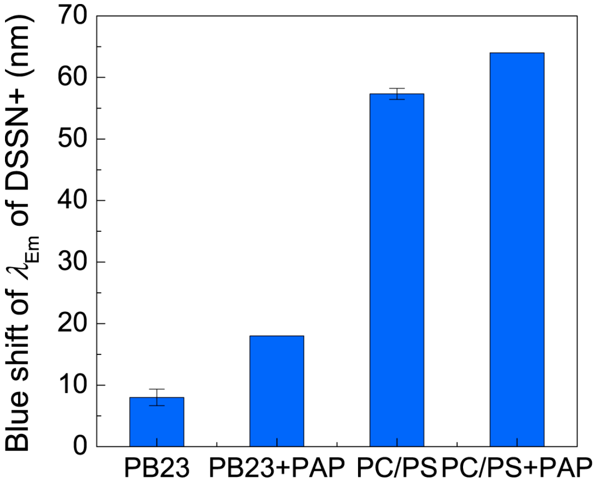

Supplementary Figure 11. Blue shift of the emission of the water soluble oligoelectrolyte DSSN+ after insertion into PB23 polymersomes, PC/PS liposomes, PAP channel incorporated PB23 polymersomes at an mCPR of 0.005 and PAP channel incorporated PC/PS liposomes at an mCLR of 0.005. The blue shift indicated a lower-polarity environment in the bilayer structures than in aqueous solution. Data shown are the average of triplicate measurements with standard deviation.

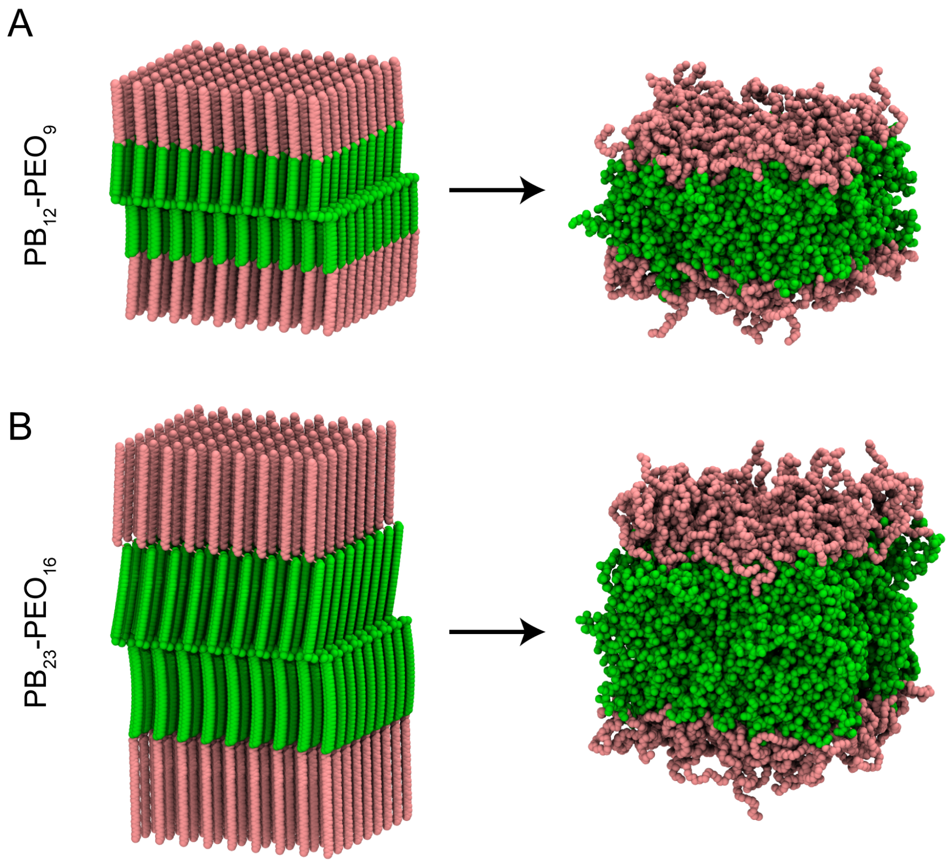

Supplementary Figure 12. Snapshots depicting initial and final equilibrated configurations of PB_{12}-PEO_9 (A) and PB_{23}-PEO_{16} (B) membranes. The hydrophobic PB chains are shown in green spheres, while the hydrophilic PEO chains are shown in pink spheres. Hydrogen atoms and water molecules have been omitted for clarity.

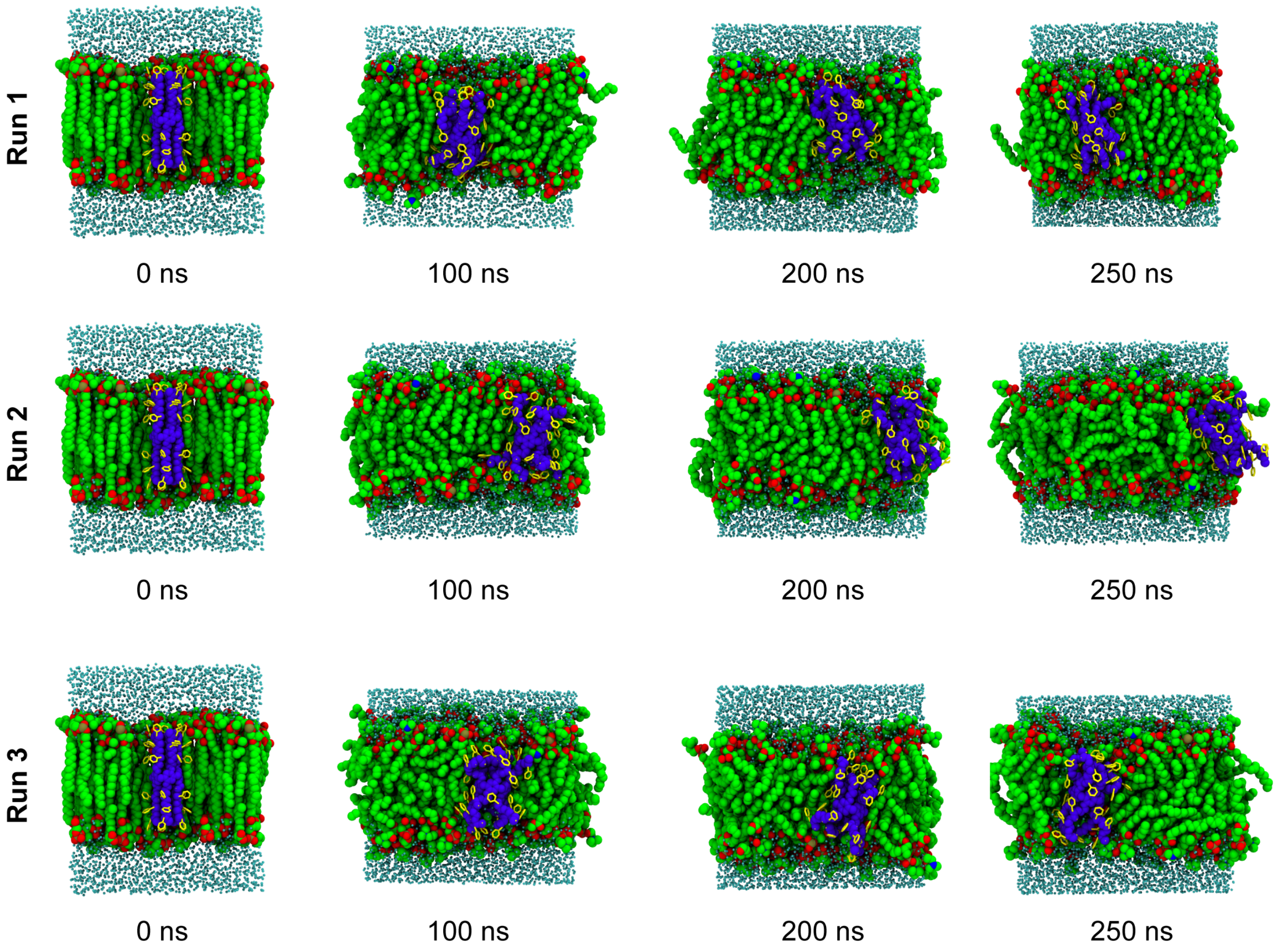

Supplementary Figure 13. Snapshots of three PAP-embedded POPC simulations are shown. The channel is depicted as a blue surface while the membrane and water molecules are shown in green and cyan wireframe representations, respectively. Each row contains four snapshots representing the beginning to the end of each simulation.

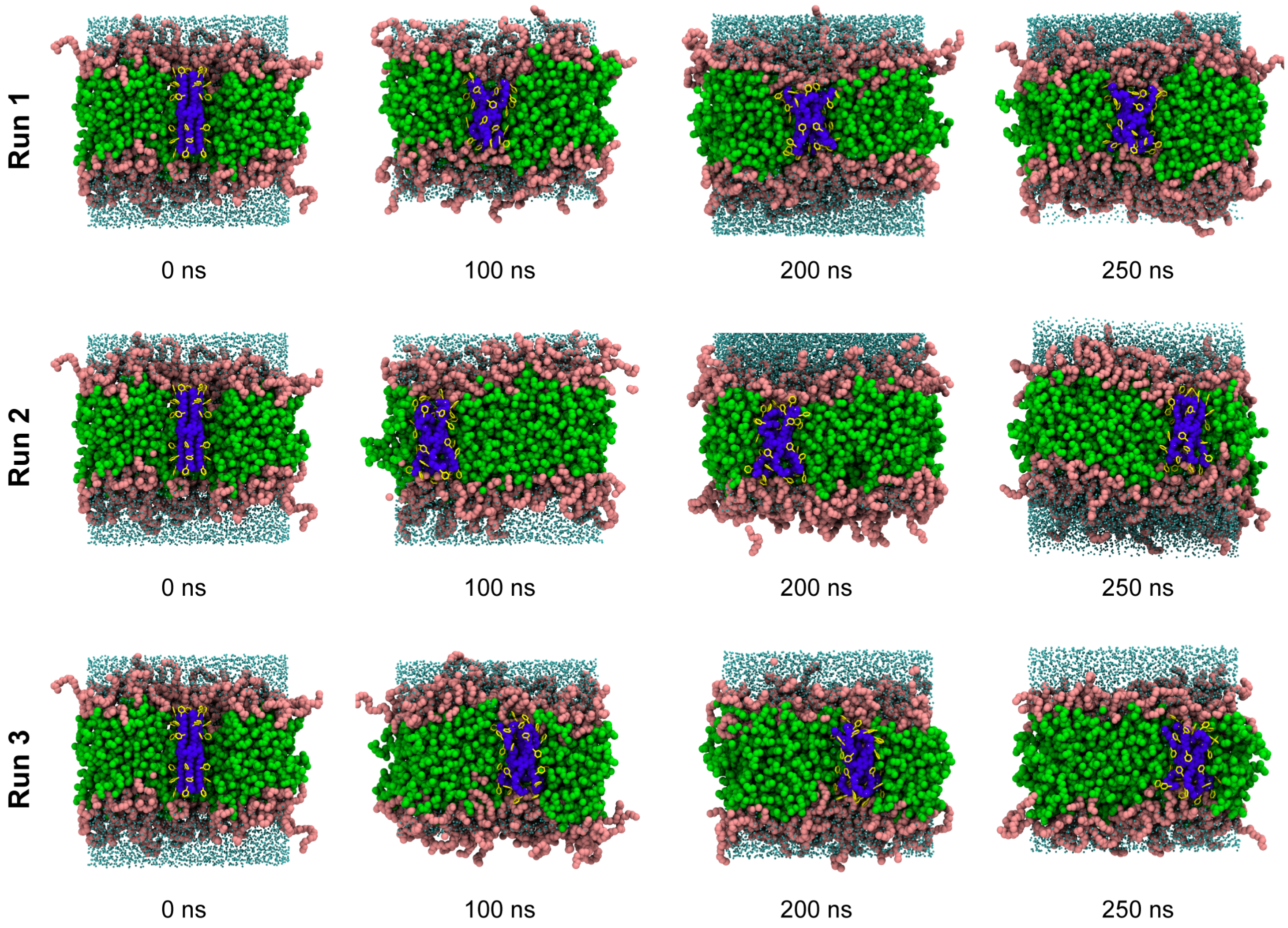

Supplementary Figure 14. Snapshots of three PAP-embedded PB12 simulations are shown. Other details are same in Supplementary Fig. 13 except that the PB and PEO chains are shown in green and pink space-filling representations.

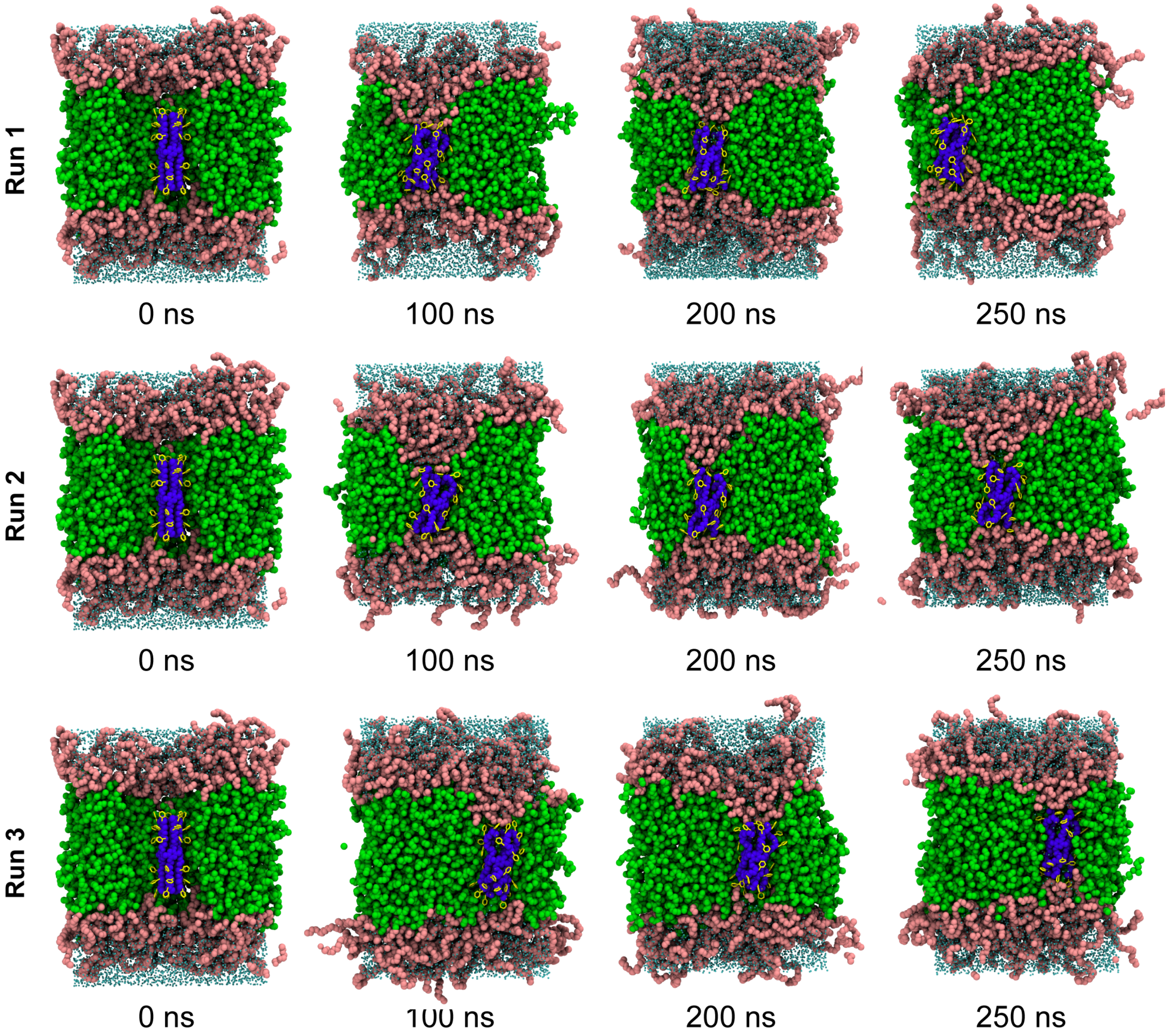

Supplementary Figure 15. Snapshots of three PAP-embedded PB23 simulations are shown. Other details are same in Supplementary Fig. 13 except that the PB and PEO chains are shown in green and pink space-filling representations. Although the length of the PAP channel is shorter than the PB23 bilayer thickness, the PAP channel in each simulation spanned the membrane.

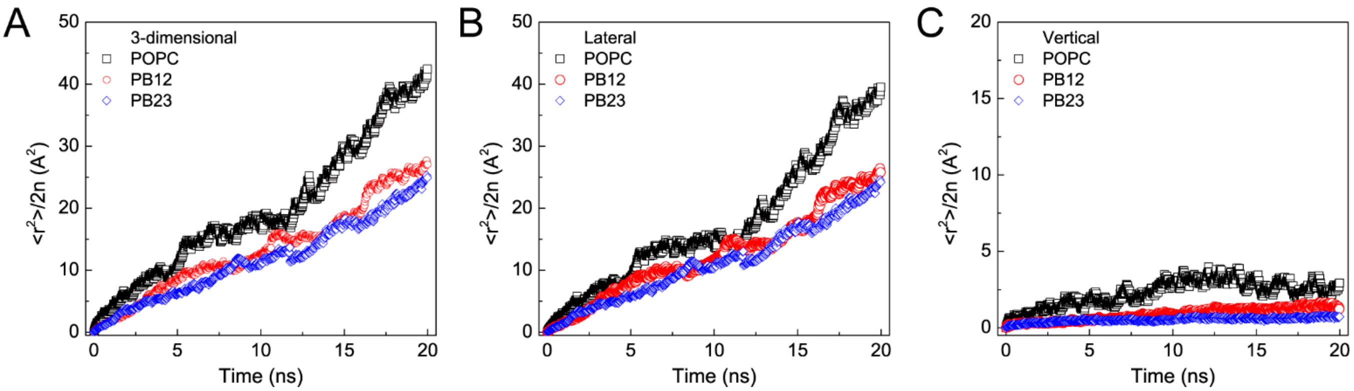

Supplementary Figure 16. The mean-squared-displacement (MSD) of the PAP channel diffusion in the three-dimensional (xyz, panel A), lateral (in xy plane, panel B), and vertical (along z-direction, panel C) directions shows that the lateral diffusion was dominant. The diffusivity coefficient in each case is equal to the slope of the line.

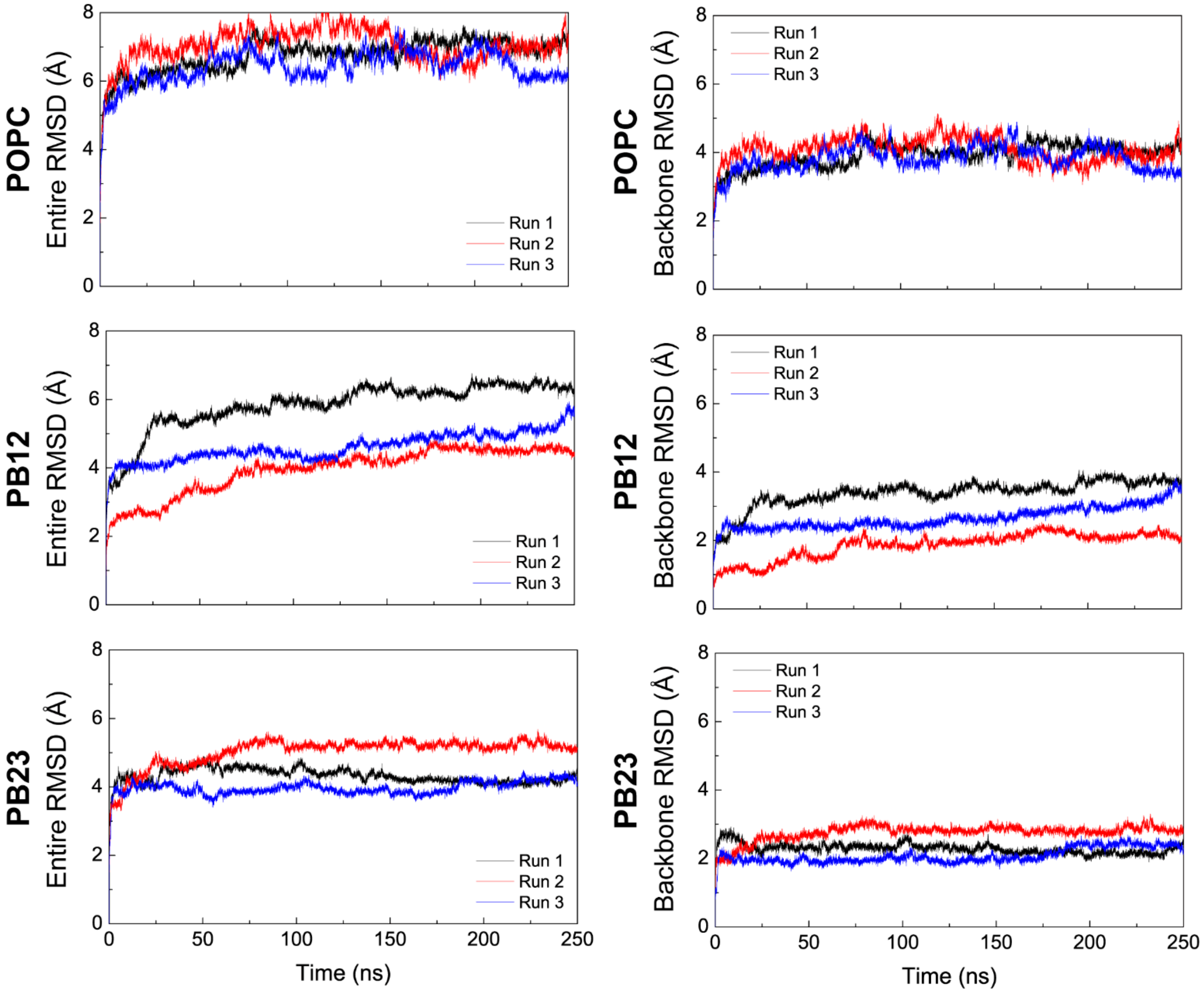

Supplementary Figure 17. Root-mean-squared-deviation (RMSD) of the PAP channel in POPC and PB-PEO membranes. Left panel shows the entire RMSD and the right panel shows the backbone RMSD.

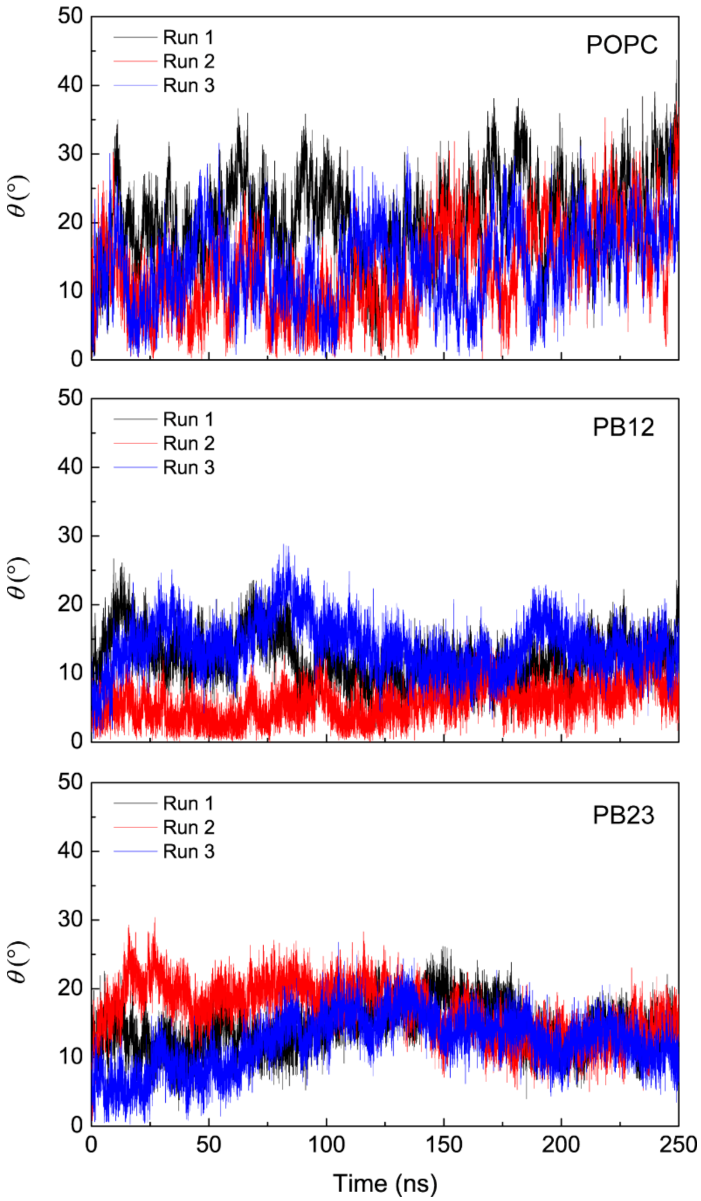

Supplementary Figure 18. The angle of orientation of the PAP channel in POPC and PB-PEO membranes. Perfect alignment of the channel axis along the z-direction corresponds to θ=0°.

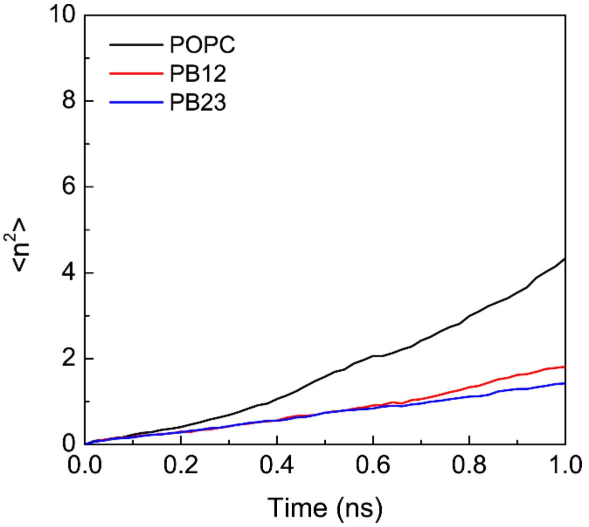

Supplementary Figure 19. MSD of the collective diffusion coordinate n against simulation time-blocks of 1 ns. The permeability of the channel is proportional to the slope of each line.

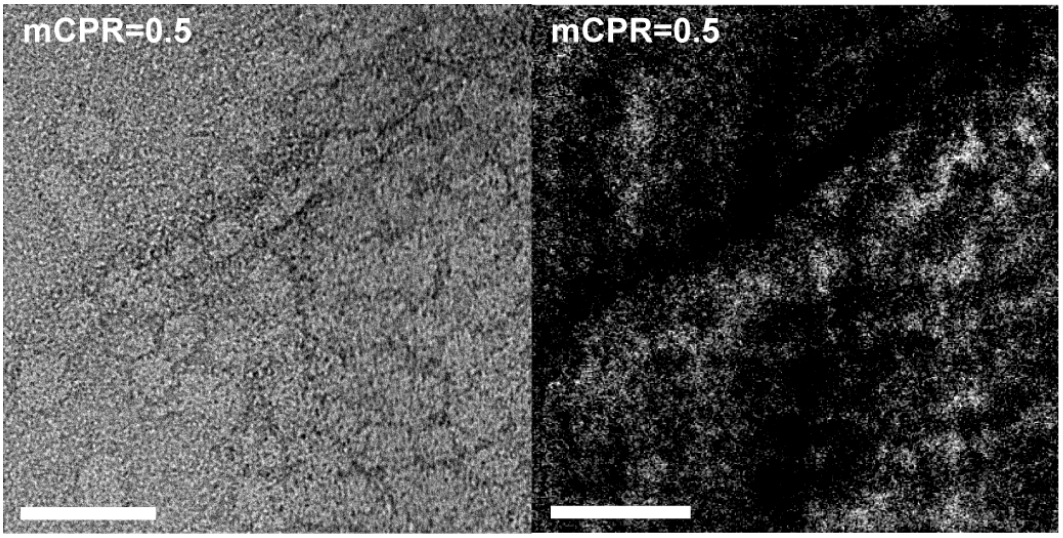

Supplementary Figure 20. Energy filtered TEM (right) of self-assembled PAP/PB12 membranes detected relatively enriched nitrogen signals from PAP channels that matched the micro-phase domains showed in the bright field image (left). Scale bar, 50 nm.

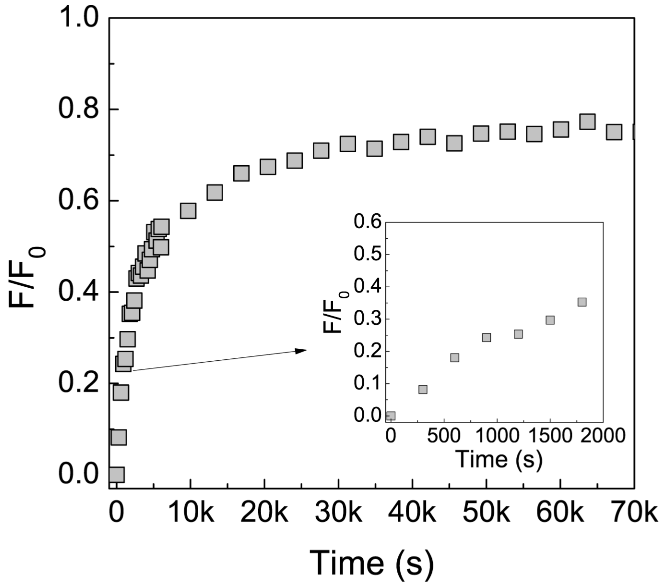

Supplementary Figure 21. The FRAP recovery kinetics of the fluorescent labeled PAP channels in PB22 polymersomes at an molar channel-to-polymer ratio of 0.01 (mCPR=0.01). We used PB22 instead of PB23 because we consumed all the original PB23 BCPs stock.

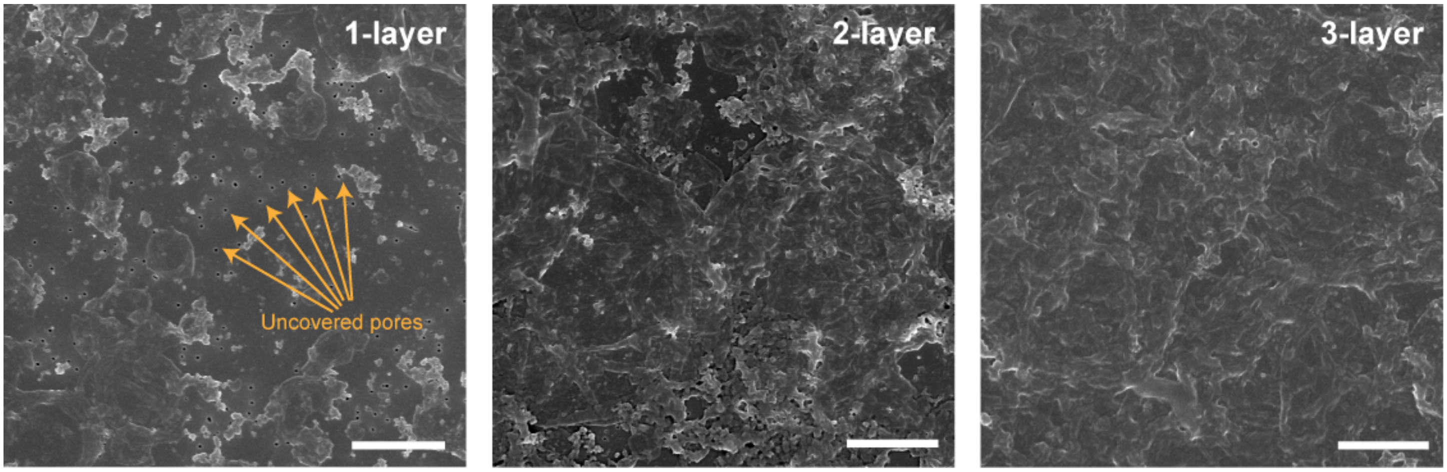

Supplementary Figure 22. SEM images showed the coverage of PAP[5] 2D sheets on PC membranes after 1, 2 and 3 cycles of layer-by-layer deposition. Scale bar, 2 μm.

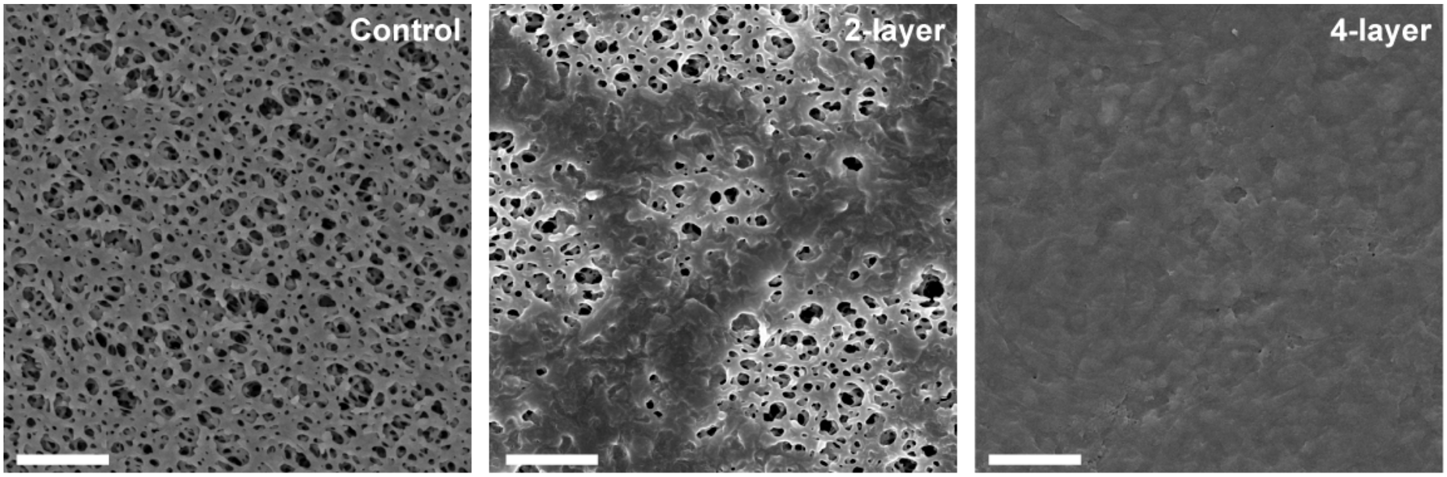

Supplementary Figure 23. SEM images showed the coverage of PAP[5] 2D sheets on PES membranes after 0, 2 and 4 cycles of layer-by-layer deposition. Scale bar, 2 μm.

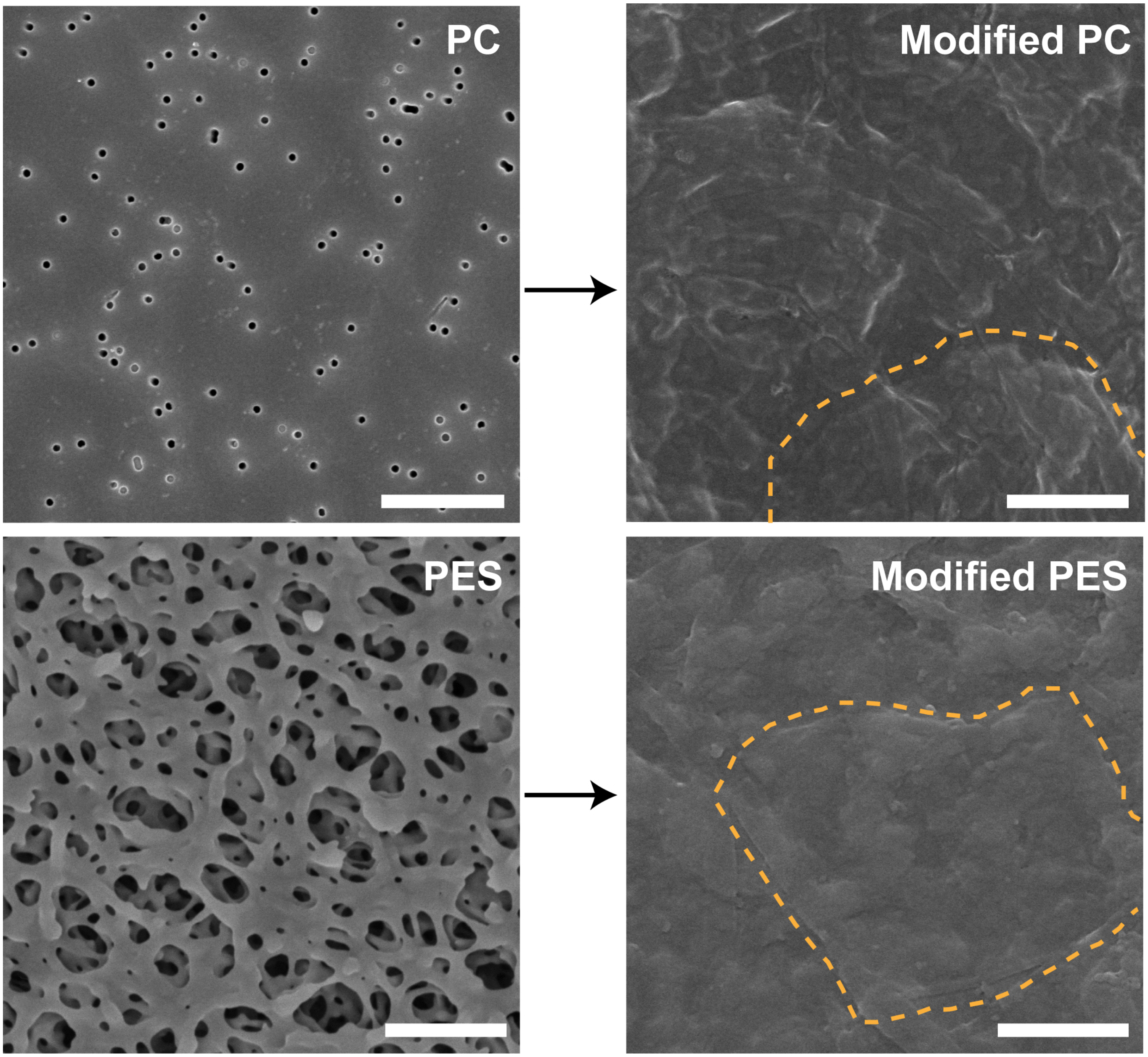

Supplementary Figure 24. SEM images showed approximately 100% coverage of PAP[5] 2D sheets on PC and PES membranes after 3 and 4 cycles of layer-by-layer deposition, respectively. The dot line indicated PAP[5] 2D sheets. Sale bar, 1 μm.

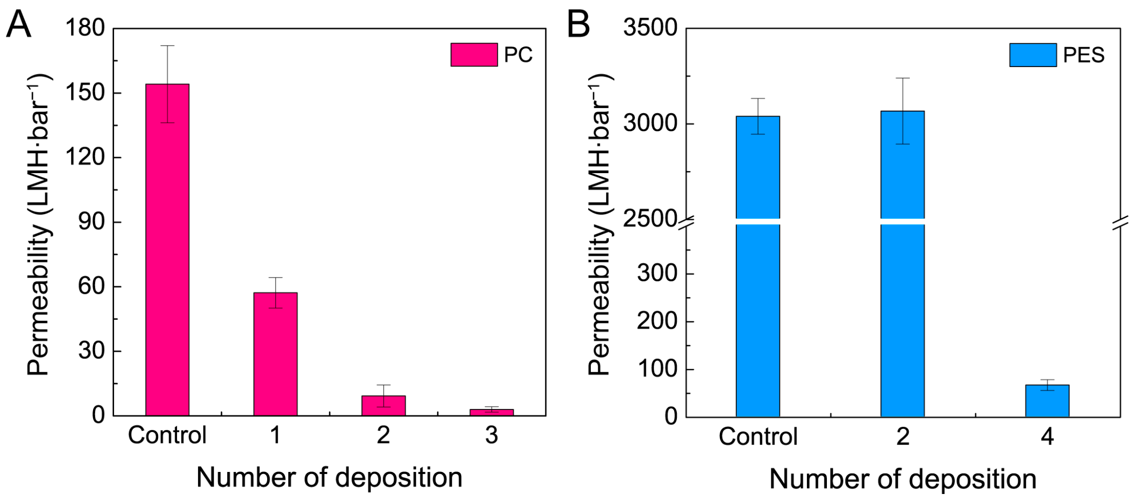

Supplementary Figure 25. For PAP[5] channels 2D sheet composite membranes, the permeability decreased as the number of depositions increased. Panel A and B were the results using 50 nm track-etched polycarbonate (PC) membranes and 30 nm polysulfone (PES) ultrafiltration membranes as support membranes, respectively. Data shown are the average of triplicate measurements with standard deviation.

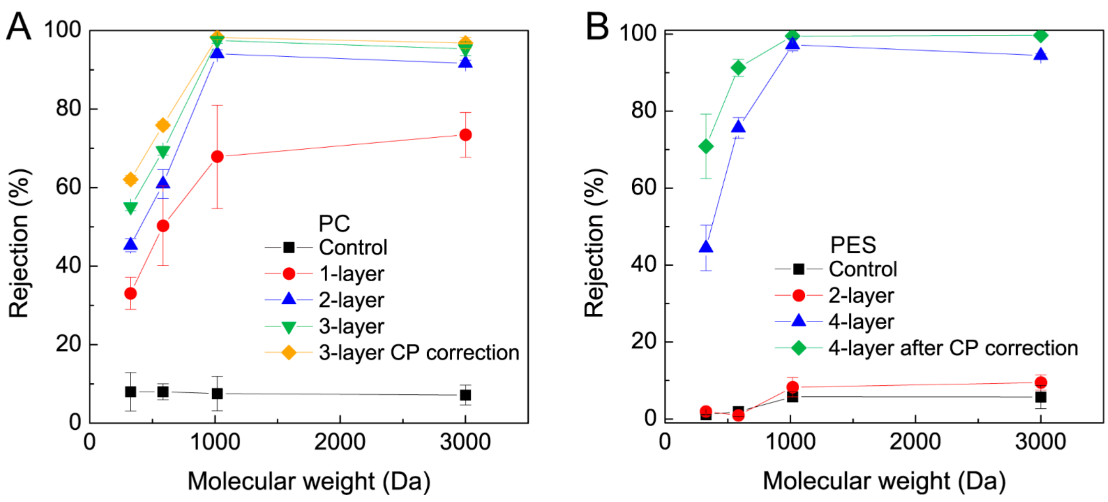

Supplementary Figure 26. For PAP[5] channels 2D sheet composite membranes, the rejection properties against dye molecules improved as the number of depositions increased. The dye molecules included methyl orange, acid fuchsin, rose bengal and fluorescent dextran. Panel A and B were the results using 50 nm track-etched polycarbonate (PC) membranes and 30 nm polysulfone (PES) ultrafiltration membranes as support membranes, respectively. The rejections (3-layer on PC and 4-layer on PES membranes) were corrected by a concentration polarization model described in Supplementary Methods. Data shown are the average of triplicate measurements with standard deviation.

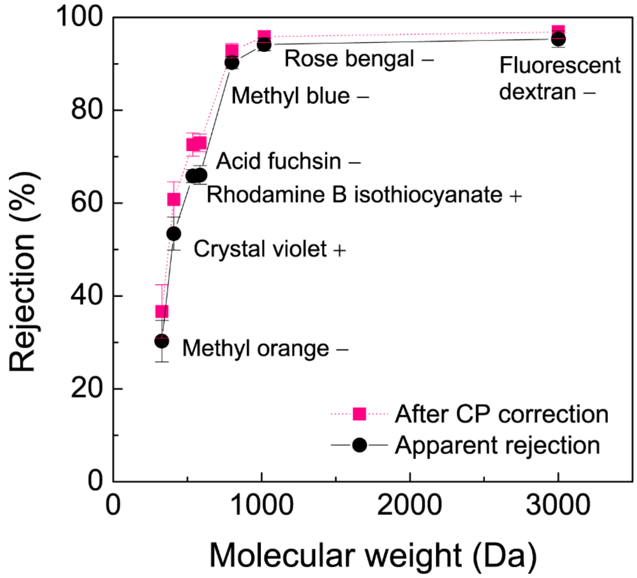

Supplementary Figure 27. For PC membranes with 3-layer PAP[5] channels 2D sheet depositions, the rejection properties (both apparent rejection and the rejection after corrected by proposed concentration polarization model) showed a consistent molecular weight cutoff trend to different charged dyes. Data shown are the average of triplicate measurements with standard deviation.

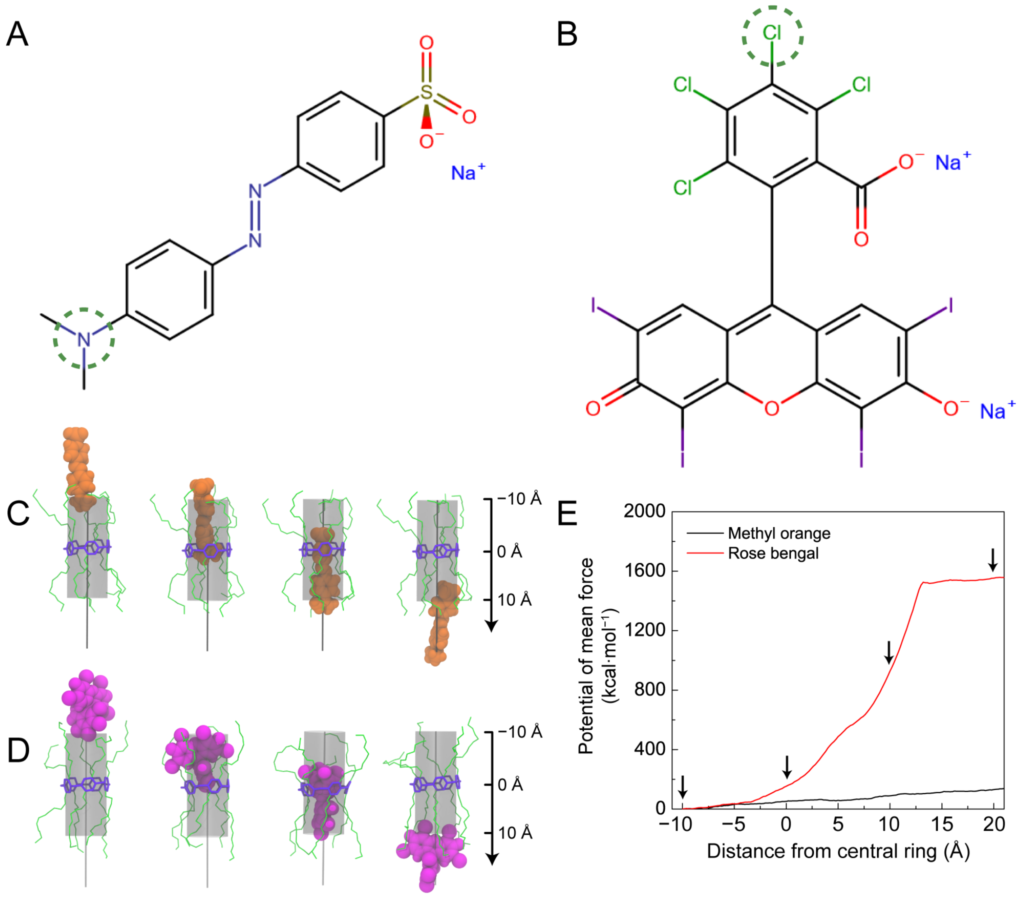

Supplementary Figure 28. A series of steered molecular dynamics simulation demonstrated the rejection behavior of PAP channels. The pulled atom in each structure (A) methyl orange (MO) and (B) rose bengal (RB) is identified by a green circle. Snapshots of MO (C) and RB (D) are shown at different points during this pulling simulation. The snapshots from left to right correspond to the locations where the pulled atoms of MO and RB (as shown in panels A and B) were −10, 0, 10, and 20 Å away from the central ring of PAP channels along the pulling trajectory. (E) The potential of mean force for pulling molecules of MO and RB through PAP demonstrated that MO could transport through PAP channels while BR should be rejected. The arrows correspond to the snapshots from left to right in panels C and D.

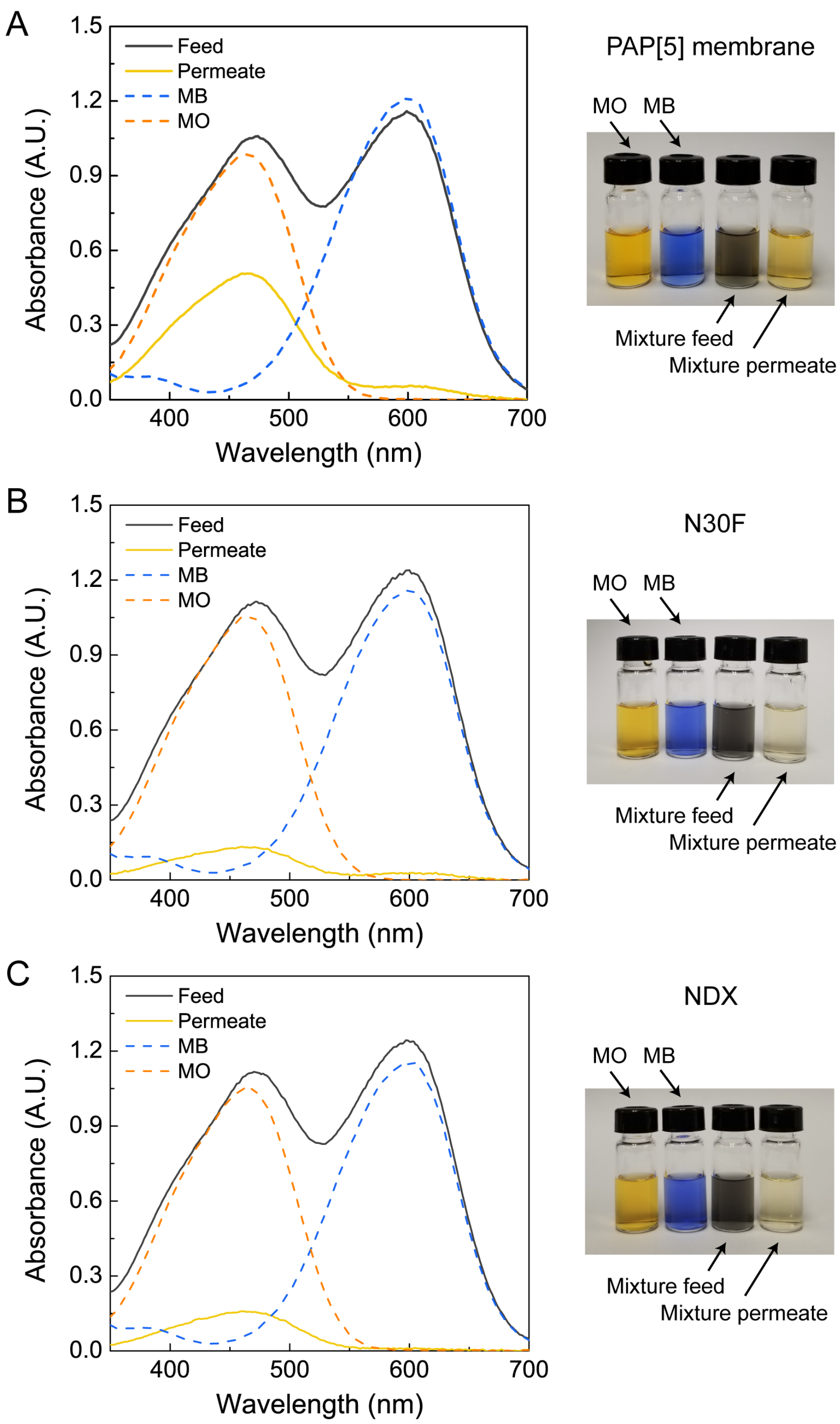

Supplementary Figure 29. Mixed feed filtration experiment with feed solutions containing two dyes (methyl blue (MB), 800 Da and methyl orange (MO), 328 Da) showed complete rejection of methyl blue while showing ~50% passage of methyl orange for (A) PC membranes with 3-layer PAP[5] channels 2D sheet deposition. For commercial membranes N30F (B) and NDX (C), these membranes almost rejected both two dyes completely although their molecular weight cutoffs are 400-500 Da.

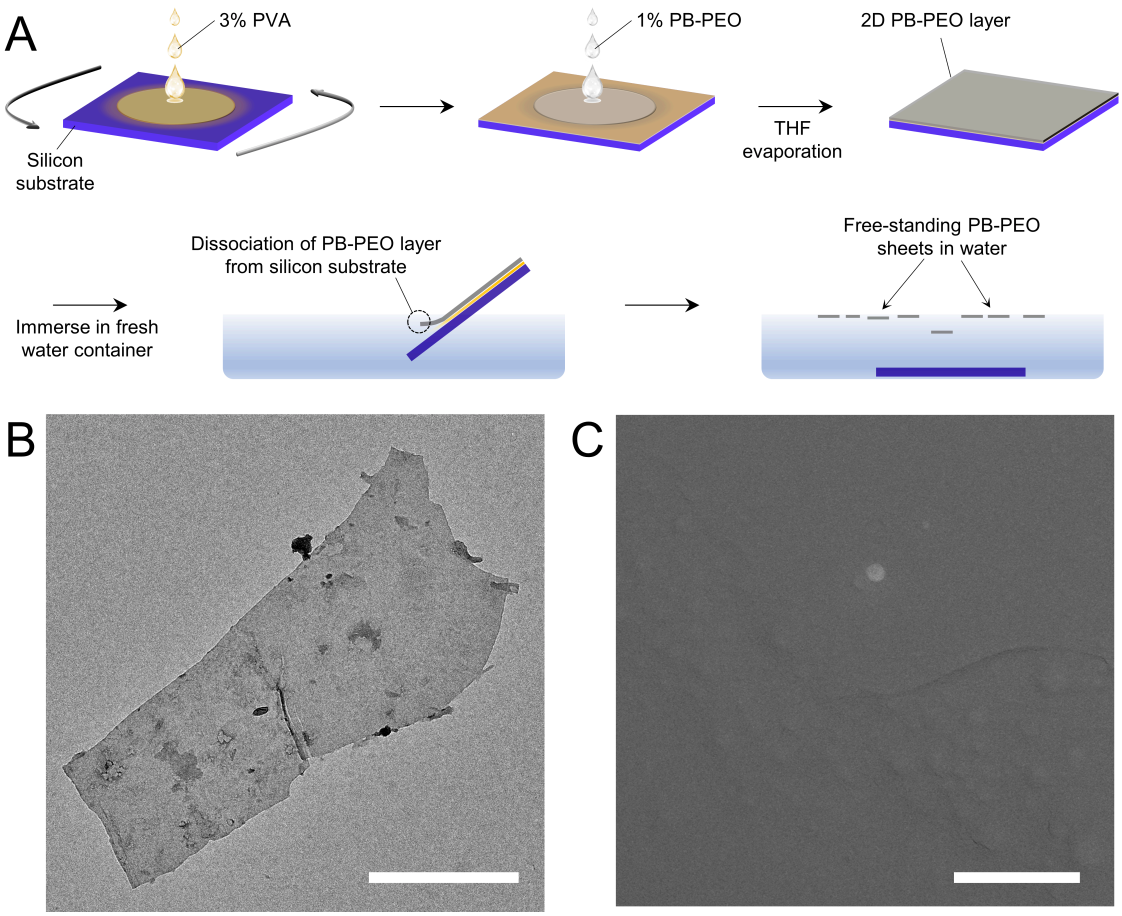

Supplementary Figure 30. PB-PEO block copolymer control membrane fabrication was based on a solvent cast method to create nanosheets that were then assembled into membranes using the layer-by-layer technique described for the PAP-based membranes. The control membrane displayed remarkably low water permeability (0.10±0.02 LMH·bar^{-1}) and significantly high rejection of methyl orange dye (95.8±0.5%, 328 Da). (A) Schematic approach of the main steps of fabricating free-standing PB-PEO 2D sheets using a sacrificial poly(vinyl alcohol) (PVA) layer. First, a PVA solution is spin coated onto the UV/ozone cleaned silicon substrate. The PB-PEO polymer solution is then drop-casted on the top of the PVA layer. The PB-PEO layer is annealed during the slow evaporation of THF followed. After solvent evaporation, the silicon substrate was gently immersed into fresh water container, allowing the casted PB-PEO sheets to be floated on water and then exploited for membrane fabrication. (B) A representative TEM image of 2D PB-PEO sheets prepared by the solvent casting method. Scale bar, 1 µm. (C) A representative SEM image of PB-PEO control membrane prepared on polyethersulfone membrane support. Scale bar, 5 µm.

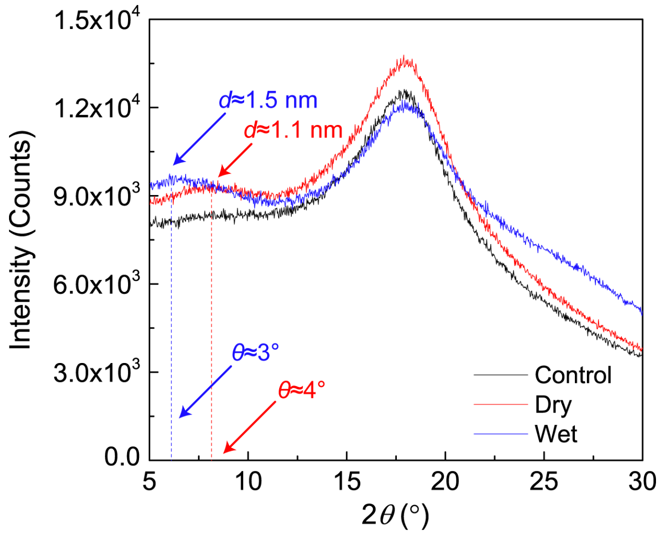

Supplementary Figure 31. X-ray diffraction patterns of control PES membrane, dry PAP[5] 2d sheet based membrane and wet PAP[5] 2d based membrane.

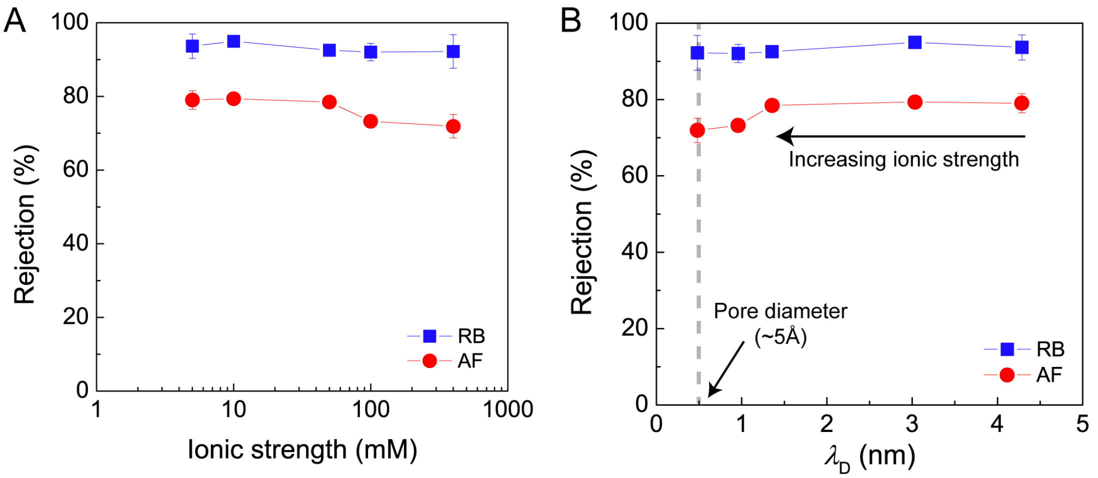

Supplementary Figure 32. Rejection test of rose bengal (RB) and acid fuchsin (AF) under various background electrolyte environments. RB (1,018Da) and AF (585 Da) were selected as solutes because their molecular weights are comparable to the MWCO of the channel based membranes (~500 Da). The ionic strength of solutions was adjusted from 5 mM to 400 mM using NaCl. The solute rejection results are plotted versus (A) the ionic strength (mM) and (B) the Debye length (λ_D, nm) of the electrolyte solutions. Data shown are the average of triplicate measurements with standard deviation.

Supplementary References

1 Chen, L. *et al.* Chiral Selective Transmembrane Transport of Amino Acids through Artificial Channels. *J. Am. Chem. Soc.* **135**, 2152-2155, (2013).

2 Hillmyer, M. A. & Bates, F. S. Synthesis and Characterization of Model Polyalkane−Poly(ethylene oxide) Block Copolymers. *Macromolecules* **29**, 6994-7002, (1996).

3 Woodle, M. C. & Papahadjopoulos, D. in *Methods Enzymol.* Vol. Volume 171 (ed Becca Fleischer Sidney Fleischer) 193-217 (Academic Press, 1989).

4 Latimer, P. & Pyle, B. E. Light Scattering at Various Angles: Theoretical Predictions of the Effects of Particle Volume Changes. *Biophys. J.* **12**, 764-773, (1972).

5 Borgnia, M. J. *et al.* Functional reconstitution and characterization of AqpZ, the E-coli water channel protein. *J. Mol. Biol.* **291**, 1169-1179, (1999).

6 Shen, Y.-x. *et al.* Highly permeable artificial water channels that can self-assemble into two-dimensional arrays. *Proc. Natl. Acad. Sci. U.S.A.* **112**, 9810-9815, (2015).

7 Erbakan, M. *et al.* Molecular Cloning, Overexpression and Characterization of a Novel Water Channel Protein from *Rhodobacter sphaeroides*. *PLoS ONE* **9**, e86830, (2014).

8 Gullapalli, R. R. *et al.* Integrated multimodal microscopy, time-resolved fluorescence, and optical-trap rheometry: toward single molecule mechanobiology. *J. Biomed. Opt.* **12**, 014012, (2007).

9 Xue, M. *et al.* Pillararenes, A New Class of Macrocycles for Supramolecular Chemistry. *Acc. Chem. Res.* **45**, 1294-1308, (2012).

10 Lopez Mora, N. *et al.* Preparation of size tunable giant vesicles from cross-linked dextran(ethylene glycol) hydrogels. *Chem. Commun.* **50**, 1953-1955, (2014).

11 Cras, J. J. *et al.* Comparison of chemical cleaning methods of glass in preparation for silanization. *Biosens. Bioelectron.* **14**, 683-688, (1999).

12 Kumar, M. *et al.* High-Density Reconstitution of Functional Water Channels into Vesicular and Planar Block Copolymer Membranes. *J. Am. Chem. Soc.* **134**, 18631-18637, (2012).

13 Axelrod, D. *et al.* Mobility measurement by analysis of fluorescence photobleaching recovery kinetics. *Biophys. J.* **16**, 1055-1069, (1976).

14 Bermudez, H. *et al.* Molecular Weight Dependence of Polymersome Membrane Structure, Elasticity, and Stability. *Macromolecules* **35**, 8203-8208, (2002).

15 Ortiz, V. *et al.* Dissipative Particle Dynamics Simulations of Polymersomes. *J. Phys. Chem. B* **109**, 17708-17714, (2005).

16 Won, Y.-Y. *et al.* Cryogenic Transmission Electron Microscopy (Cryo-TEM) of Micelles and Vesicles Formed in Water by Poly(ethylene oxide)-Based Block Copolymers. *J. Phys. Chem. B* **106**, 3354-3364, (2002).

17 Srinivas, G. *et al.* Self-assembly and properties of diblock copolymers by coarse-grain molecular dynamics. *Nat Mater* **3**, 638-644, (2004).

18 Kale, L. *et al.* NAMD2: Greater scalability for parallel molecular dynamics. *J Comput Phys* **151**, 283-312, (1999).

19 Humphrey, W. *et al.* VMD: Visual molecular dynamics. *J. Mol. Graphics* **14**, 33-38, (1996).
20 MacKerell, A. D. *et al.* All-atom empirical potential for molecular modeling and dynamics studies of proteins. *The journal of physical chemistry. B* **102**, 3586-3616, (1998).
21 Zhu, F. *et al.* Collective Diffusion Model for Water Permeation through Microscopic Channels. *Phys. Rev. Lett.* **93**, 224501, (2004).
22 Rathee, V. S. *et al.* A coarse-grained thermodynamic model for the predictive engineering of valence-selective membranes. *Molecular Systems Design & Engineering* **1**, 301-312, (2016).
23 Seonghoon, K. *et al.* CHARMM‐GUI ligand reader and modeler for CHARMM force field generation of small molecules. *J. Comput. Chem.* **38**, 1879-1886, (2017).
24 Grzelakowski, M. *et al.* A framework for accurate evaluation of the promise of aquaporin based biomimetic membranes. *J. Membr. Sci.* **479**, 223-231, (2015).
25 Rajesh, S. *et al.* Mixed Mosaic Membranes Prepared by Layer-by-Layer Assembly for Ionic Separations. *ACS Nano* **8**, 12338-12345, (2014).
26 Lang, C. *et al.* Creating Cross-Linked Lamellar Block Copolymer Supporting Layers for Biomimetic Membranes. *Faraday Discuss.*, (2018).
27 Mo, Y. *et al.* Cation-dependent structural instability of graphene oxide membranes and its effect on membrane separation performance. *Desalination* **399**, 40-46, (2016).
28 Zydney, A. L. Stagnant film model for concentration polarization in membrane systems. *J. Membr. Sci.* **130**, 275-281, (1997).
29 Feroz, H. *et al.* Concentrating membrane proteins using ultrafiltration without concentrating detergents. *Biotechnol. Bioeng.* **113**, 2122-2130, (2016).
30 Colton, C. K. & Smith, K. A. Mass transfer to a rotating fluid. Part II. Transport from the base of an agitated cylindrical tank. *AlChE J.* **18**, 958-967, (1972).
31 Koutsou, C. P. & Karabelas, A. J. Shear stresses and mass transfer at the base of a stirred filtration cell and corresponding conditions in narrow channels with spacers. *J. Membr. Sci.* **399**, 60-72, (2012).
32 Erickson, H. P. Size and Shape of Protein Molecules at the Nanometer Level Determined by Sedimentation, Gel Filtration, and Electron Microscopy. *Biological Procedures Online* **11**, 32-51, (2009).
33 Dean, D. C. *et al.* Modeling healthy male white matter and myelin development: 3 through 60 months of age. *NeuroImage* **84**, 742-752, (2014).
