# Supplementary material for: Achieving high permeability and enhanced selectivity for Angstrom-scale separations using artificial water channel membranes
Source: Nat Commun. 2018 Jun 12;9:2294. doi: 10.1038/s41467-018-04604-y (PMC5997692; doi:10.1038/s41467-018-04604-y)
Supplement: Supplementary file 2 — Description of Additional Supplementary Files [file 41467_2018_4604_MOESM2_ESM.pdf]

Description of Additional Supplementary Files

File Name: Supplementary Movie 1
Description: Movie of a single methyl orange (orange molecule) SMD simulation, paired with the plot of the averaged force vs. distance from this SMD pulling simulation. The molecules were originally placed 10 Å from the central ring. The movie represents 5 ns of data and the molecule is pulled at a rate of 10 Å·ns−1 with a spring constant of 1000 kcal·mol−1. Harmonic constraints with a force constant of 2 kcal·mol−1 were kept on the central ring of PAP (purple) to hold it in place. The PAP backbone is shown in green. From the simulation, we observed no deformation of either methyl orange molecule or the PAP5 central ring, indicating that there is no significant energy barrier for this molecule to pass through the channel.

File Name: Supplementary Movie 2
Description: Movie of a single rose bengal (magenta molecule) SMD simulation, paired with the plot of the averaged force vs. distance from this SMD pulling simulation. The molecules were originally placed 10 Å from the central ring. The movie represents 5 ns of data and the molecule is pulled at a rate of 10 Å·ns−1 with a spring constant of 1000 kcal·mol−1. Harmonic constraints with a force constant of 2 kcal·mol−1 were kept on the central ring of PAP (purple) to hold it in place. The PAP backbone is shown in green. From the simulation, we observed deformation of both the methyl orange molecule as well as the PAP5 central ring, as illustrated by the two sharp jumps in the force profile. The rose bengal got stuck in the channel until the SMD dummy atom generates enough force to deform the molecule and pull it through, which would not be expected to occur naturally.
